# A network-based approach to deciphering a dynamic microbiome’s response to a subtle perturbation

**DOI:** 10.1038/s41598-020-73920-5

**Published:** 2020-11-11

**Authors:** Grace Tzun-Wen Shaw, An-Chi Liu, Chieh-Yin Weng, Yi-Chun Chen, Cheng-Yu Chen, Francis Cheng-Hsuan Weng, Daryi Wang, Chu-Yang Chou

**Affiliations:** 1 Biodiversity Research Center, Academia Sinica, Taipei, 115 Taiwan; 2 Bioenergy Research Center, National Taiwan University, Taipei, Taiwan; 3 Department of Biomechatronics Engineering, National Taiwan University, Taipei, Taiwan

**Keywords:** Biochemical reaction networks, Classification and taxonomy, Computational models, Network topology, Microbiology

## Abstract

Over the past decades, one main issue that has emerged in ecological and environmental research is how losses in biodiversity influence ecosystem dynamics and functioning, and consequently human society. Although biodiversity is a common indicator of ecosystem functioning, it is difficult to measure biodiversity in microbial communities exposed to subtle or chronic environmental perturbations. Consequently, there is a need for alternative bioindicators to detect, measure, and monitor gradual changes in microbial communities against these slight, chronic, and continuous perturbations. In this study, microbial networks before and after subtle perturbations by adding *S. acidaminiphila* showed diverse topological niches and 4-node motifs in which microbes with co-occurrence patterns played the central roles in regulating and adjusting the intertwined relationships among microorganisms in response to the subtle environmental changes. This study demonstrates that microbial networks are a good bioindicator for chronic perturbation and should be applied in a variety of ecological investigations.

## Introduction

The past decades have seen remarkable progress in understanding how human activities influence Earth’s ecosystems^1^. The loss of biological diversity, in terms of all life on earth, genetic variation among creatures, and entire ecosystems, impacts ecological processes, ecosystem services, human society, and economics. Most recently, the intertwined relationship between global environmental change and biodiversity dynamics has become a new impetus for ecological research^2,3^. Major strides have been made in rethinking how we conserve natural resources sustainably, redefining how we evaluate the quality of ecosystems, and refining biological indicators to accurately measure biodiversity^1,2,4^. A powerful bioindicator, e.g. plants, planktons, animals, and microbes, can show how quickly a natural surrounding is changing, or predict how it will change. For example, marine pollution can be detected by measuring changes in phytoplankton diversity^5,6^. The quality of aquatic and terrestrial habitats can be monitored by changes in rotifer^7^, leech^6^, and macrobenthos^8^ populations, among others. Several studies recently highlighted the importance of microorganisms for determining low levels of contaminants and small biological changes, owing to their rapid growth^9,10^.

Due to their rapid growth, easy to test and readily available, there is mounting evidence to integrate microbial biodiversity into studies of ecosystem processes or environmental changes^11^. Current negative trends in microbial biodiversity are mainly due to climbing anthropogenic pressures, e.g. resource consumption, invasive alien species, pollution, and habitat destruction, and rapid climate change^1,4^. These large perturbations directly affect microbial species, leading to compositional changes, and indirectly alter species’ behaviors and the strength of inter-species interactions, all of which result in a dynamic ecological network. However, most conventional indexes capturing taxonomic diversity based only on species abundance, richness, and evenness—such as the Shannon index^12^, Simpson index^13^, and Chao-1 index^14^—do not assess the effect of interrelatedness among species on the stability of the entire ecosystem, and therefore lack the sensitivity to respond to subtle and chronic environmental degradation.

Microbes interact with their communities in a complicated way. Using correlation, co-occurrence or co-exclusion, to measure microbial relationships^15^ is the simplest approach to potentially identifying pairs that are metabolically complementary^16^. Mutualistic microbes may benefit each other and correlate positively among samples. Competitive microbes may compete with one another, leading to a negative correlation trend^15,16^. Studies of co-occurrence networks in microbial communities have confirmed the connection between network structure and chronic (and subtle) environmental changes due to soil anthropization^17^, litter quality^18^, and air pollution^19,20^, in which microbial biodiversity, e.g. Shannon and inverse Simpson index, might therefore lead to the conclusion that the ecosystem remains unaltered if the perturbation is of subtle intensity.

Over the past decades, researchers have become increasingly aware of subtle changes in environmental conditions during studies on ecological sustainability and the impacts of anthropogenic and natural processes^11,21^. A robust microbial ecosystem has four major drivers influencing microbial biodiversity, which can be informative or sensitive indicators of an ecosystem’s response to subtle perturbations: rare species effect, resistance/resilience effect, spatial effect, and microbial interactive effect^22^. Measuring microbial diversity at different spatial and temporal scales is another way of using microorganisms as indicators. Priority effects during microbial colonization have long-lasting consequences for the development of microbial communities and constitutes a major barrier to entry for microbes entering a community; this is also called colonization resistance^23,24^.

The purpose of this study is to describe the application of microbial networks to detect a subtle and chronic environmental change. More specifically, this study was undertaken to understand how a subtle perturbation can illustrate the concept of colonization resistance using a novel network-based bioaugmentation approach in an anaerobic digestion system, and to suggest some practical implications and connections of co-occurrence patterns to future work. In this paper, we present a conceptual framework that links subtle perturbations; the unchangeableness of biodiversity; and the dynamics of microbial composition, co-occurrence, and networks. Their unique capabilities make the use of microbial networks to detect subtle perturbations much more useful than conventional biodiversity measurements.

## Results

### Creating an artificial subtle perturbation

The so-called priority effect is the impact that the first colonized microbial population has in hampering the subsequent colonization of microbial immigrants and becoming a defense mechanism that helps an ecosystem confer colonization resistance against invading bacterial pathogens^24,25^. Therefore, adding pregrown microbial cultures can disturb the microecological balance and re-establish the entire community as part of the pregrown culture’s niche. A novel way to artificially create a microbial ecosystem under pressure from subtle perturbations is to add a bioaugmented microbial species to disrupt microbiota-mediated colonization resistance transitorily was currently proposed using an anaerobic digestion system. Bioaugmented microbial species were added to the mesophilic anaerobic digesters, and their disruptive effects on the anaerobic digestion system were evaluated once the biogas and methane production rose.

**Figure 1:**
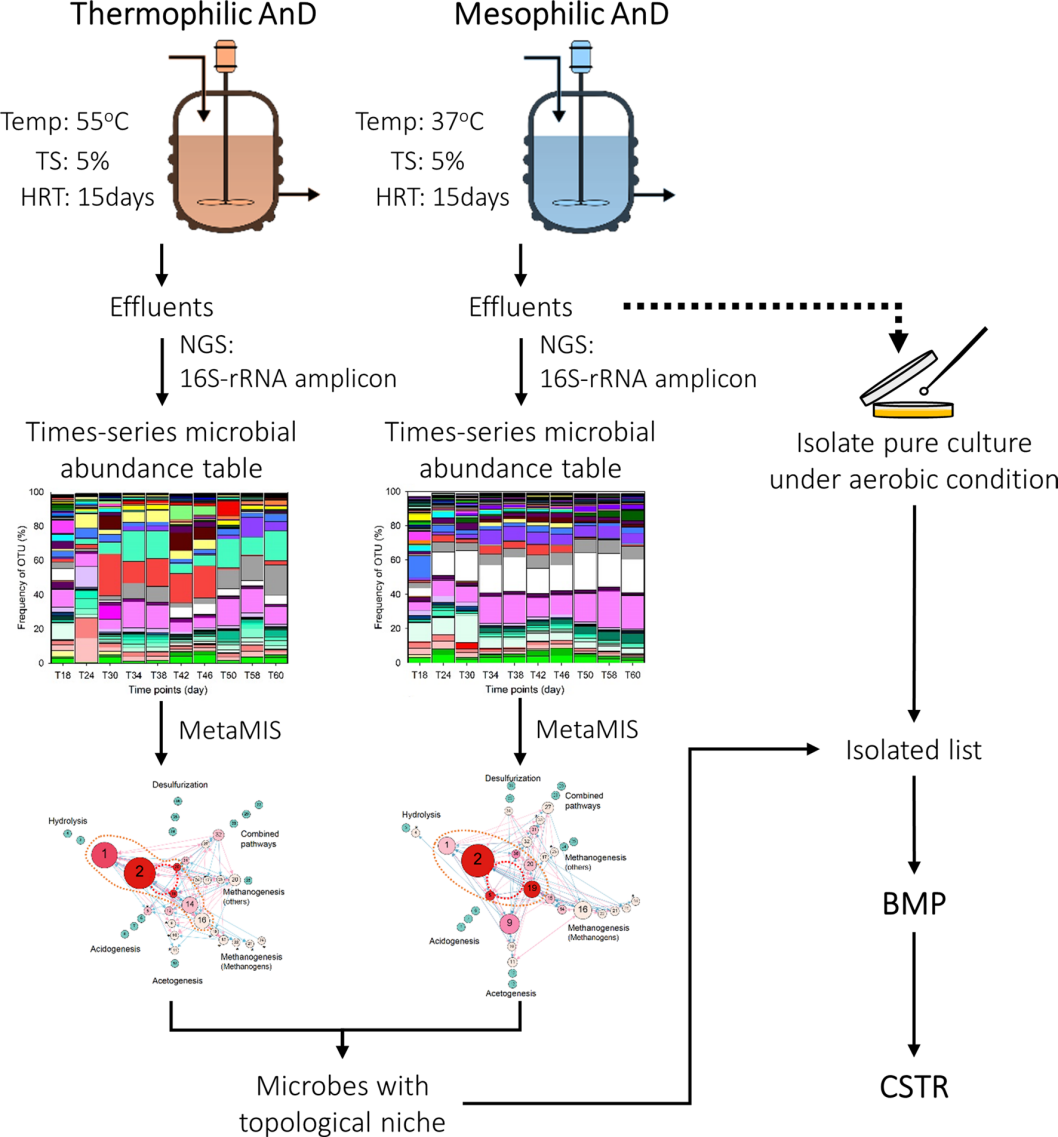
A flowchart of the network-based bioaugmentation approach. The mesophilic and thermophilic microbial networks were derived from a previous study^31^.

**Figure 2:**
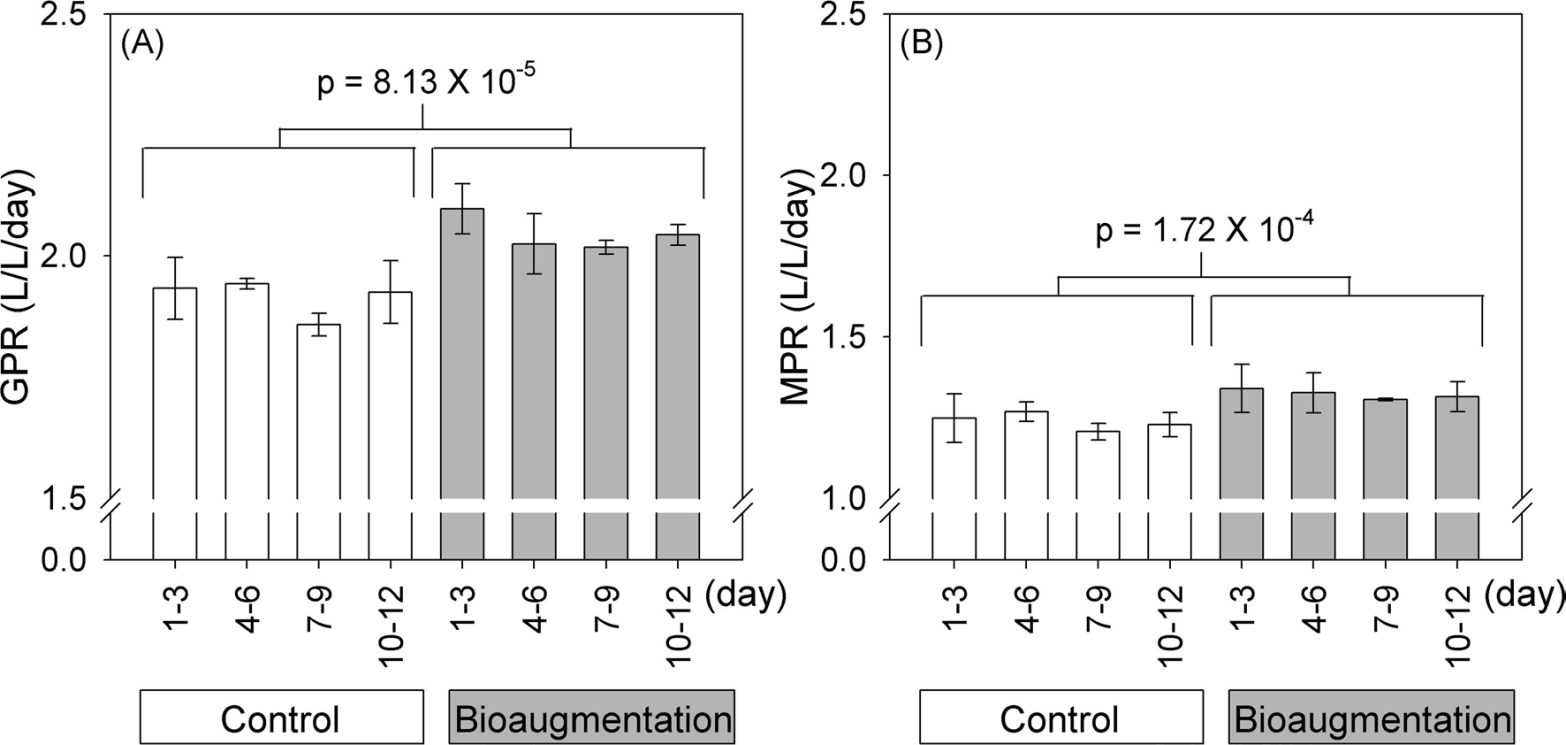
The effect of adding *S. acidaminiphila* to daily-fed CSTR. Compared to the control group, adding *S. acidaminiphila* significantly raised the level of (**A**) GPR and (**B**) MPR. Two-sample Student’s t test was used to compare the differences in GPR or MPR between the control and bioaugmentation treatments.

The supplemental microbial species added (Supplementary Tables S1 and S2) were selected based on a novel network-based approach (Fig. 1) coupled with four decisive criteria proposed by this study (For more details, see Supplementary Results 1 and Supplementary Fig. S1). To simulate an artificial subtle perturbation in a microbial ecosystem, *Stenotrophomonas acidaminiphila* (Family: *Xanthomonadaceae*) was chosen from four selected species under a quickly assessed BMP (biochemical methane potential) test (Supplementary Results 2, Supplementary Table S3) to increase biogas yields under a long-term anaerobic digestion system, called CSTR (continuous stirred tank reactors) (Fig. 2 and Supplementary Fig. S2). The bioaugmented CSTR reactors using *S. acidaminiphila* showed a significantly enhanced GPR (gas production rate) from 1.92 ± 0.02 to 2.05 ± 0.02 L/L/day (p = 8.13 $\times$ 10^–5^) and MPR (methane production rate) from 1.24 ± 0.02 to 1.32 ± 0.03 L/L/day (p = 1.72 $\times$ 10^–4^), meaning that it had successfully disrupted the stable microsystem (Fig. 2) which was hardly detected by biodiversity measurements (Supplementary Results 2). To confirm the effect of *S. acidaminiphila* on biogas yields, eight anaerobic digesters—four each for the control (C) and bioaugmentation (B) treatments—were used to reproduce the process (Supplementary Fig. S2). Therefore, a subtle perturbation was successfully performed in the laboratory scale anaerobic digestion system based on the topological niche of microbial networks, meaning that this is a novel and prominent method for identifying potential influencers that can slightly disturb a stable microecosystem.

### The effects of subtle perturbation on microbial communities

**Figure 3:**
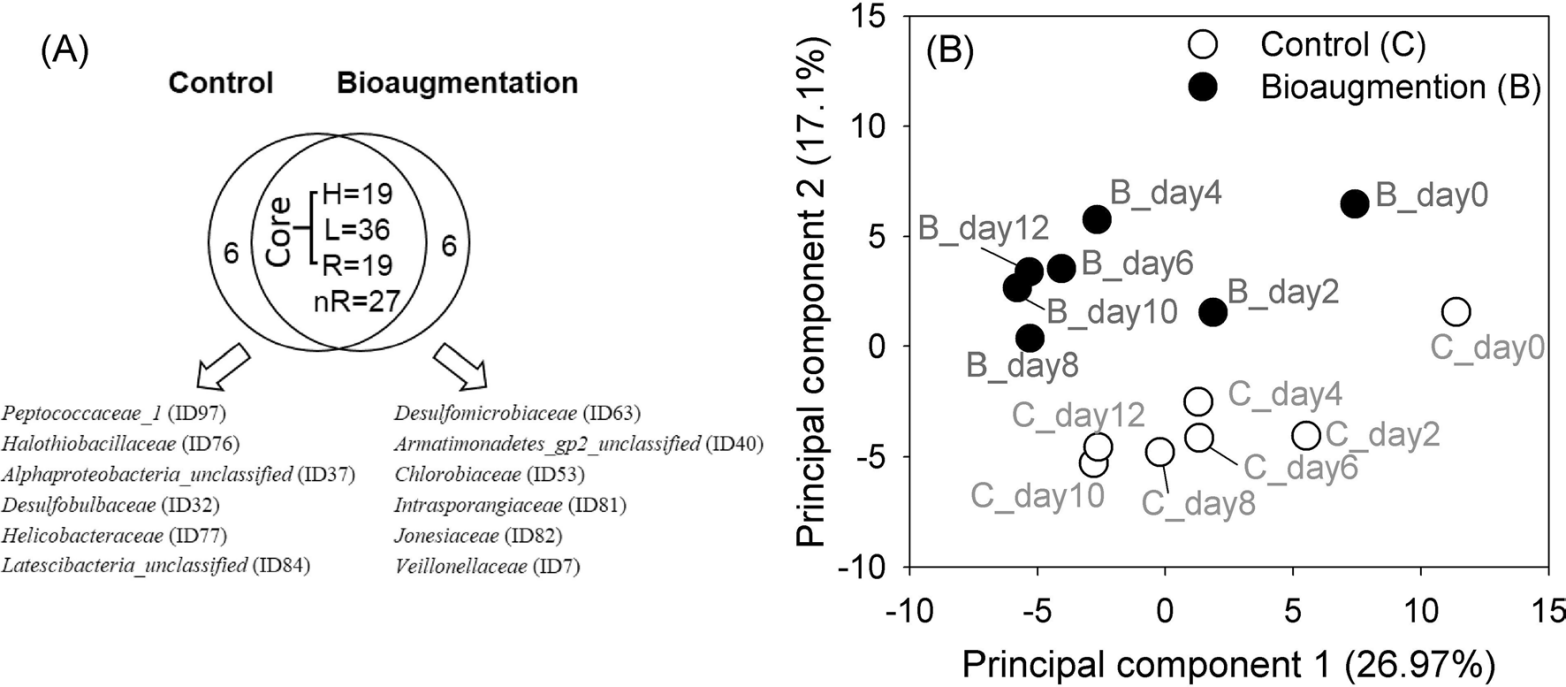
The effects of bioaugmentation on microbial communities. (**A**) Venn diagram of 113 sequenced microbes with and without bioaugmentation. (**B**) The PCA plot separated samples, with 44.07% of variance explained by the first two principal components. Families with coherent presence (Core) among samples and high, low or rare abundance were denoted as H, L or R. Families with rare abundance had some zero abundance value among samples and denoted by nR.

The next step was to identify the dynamic changes in microbial communities due to *S. acidaminiphila* perturbation that could not be detected by microbial biodiversity (Supplementary Results 2). Fourteen time-series samples from the mesophilic anaerobic digesters (CSTR) with and without the subtle perturbation were collected and manipulated by 16S rRNA amplicon sequencing analysis; after all the 16S rRNA sequence preprocessing steps were performed, 113 microbial families (Fig. 3A) were identified with an average of 19,392 ± 982 bacteria and archaea reads (Supplementary Tables S4 and S5).

When *S. acidaminiphila* was added, the microbial ecosystem preserved most of its members (101 overlapping taxa), and only a small number of rare families (12 specific taxa) emerged (Fig. 3A). This kind of conservation and stimulation in the microbial communities under pressure from subtle perturbation pushes the entire system in a discrepant situation in which the microbial diversities do not vary (Supplementary Table S6) but do segregate into two microbial communities—with and without bioaugmentation—based on principal component analysis (PCA) (Fig. 3B). The separation between microbiomes indicated that the addition of *S. acidaminiphila* changed the microbial communities of anaerobic digesters.

**Figure 4:**
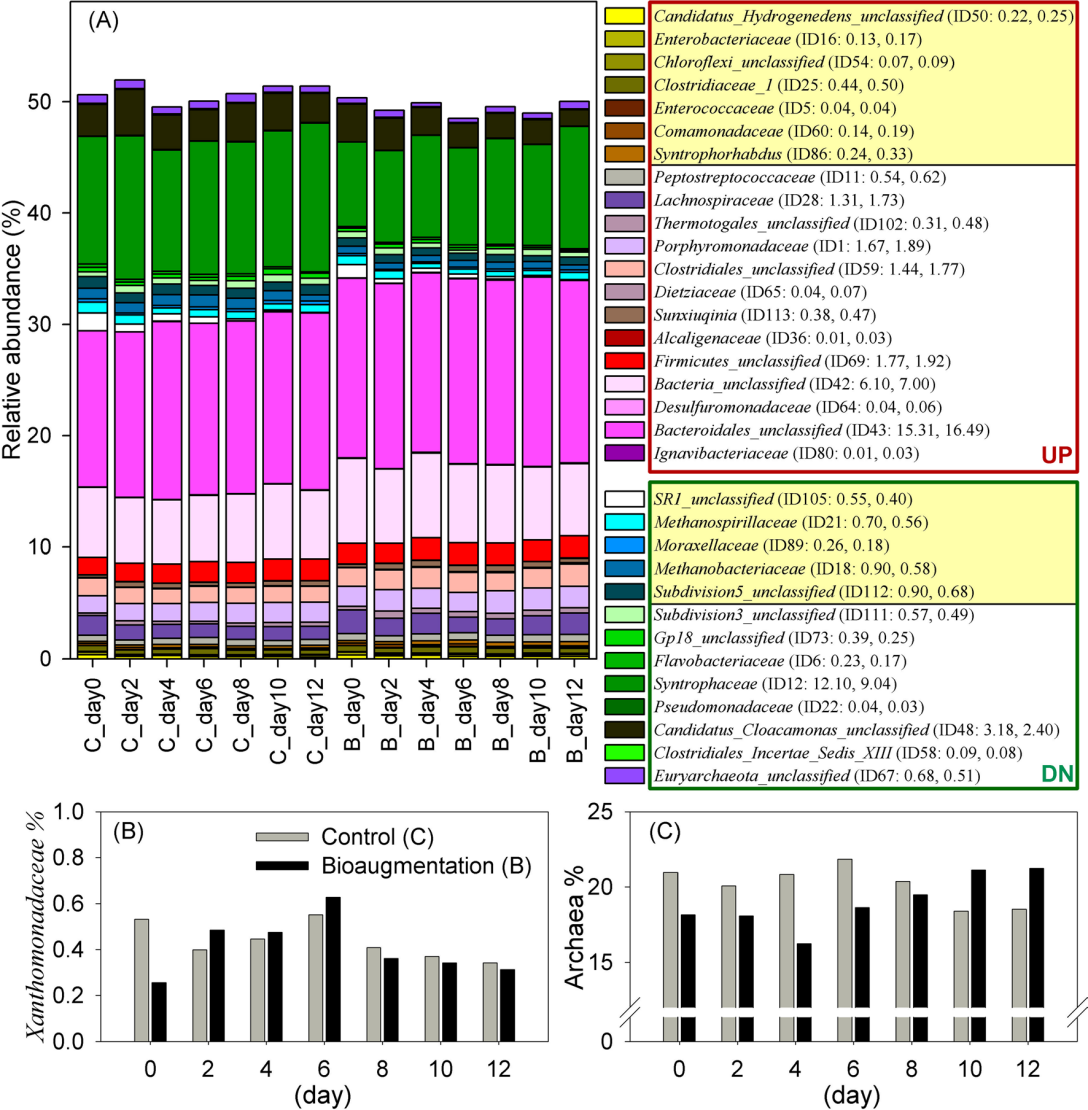
The influence of bioaugmentation on microbial communities. The abundance profiles of (**A**) 33 differential abundant families, (**B**) family *Xanthomonadaceae* (C:0.44 ± 0.03, B:0.41 ± 0.05; p = 0.576), and (**C**) the archaeal population (C:20.14 ± 0.48, B:18.99 ± 0.67; p = 0.332). The microbial population is denoted as mean and standard error. Paired-samples Student’s t test (H_0_: U_C_ = U_B_) for population comparison. Framed in red (or green) indicates increased (or decreased) abundance, denoted as UP (or DN). Colored in yellow are microbial members with co-occurrence patterns, ${\text{r}}_{{{\text{C}}_{{\text{i}}} {\text{B}}_{{\text{i}}} }}$(Fig. 5(A)).

**Figure 5:**
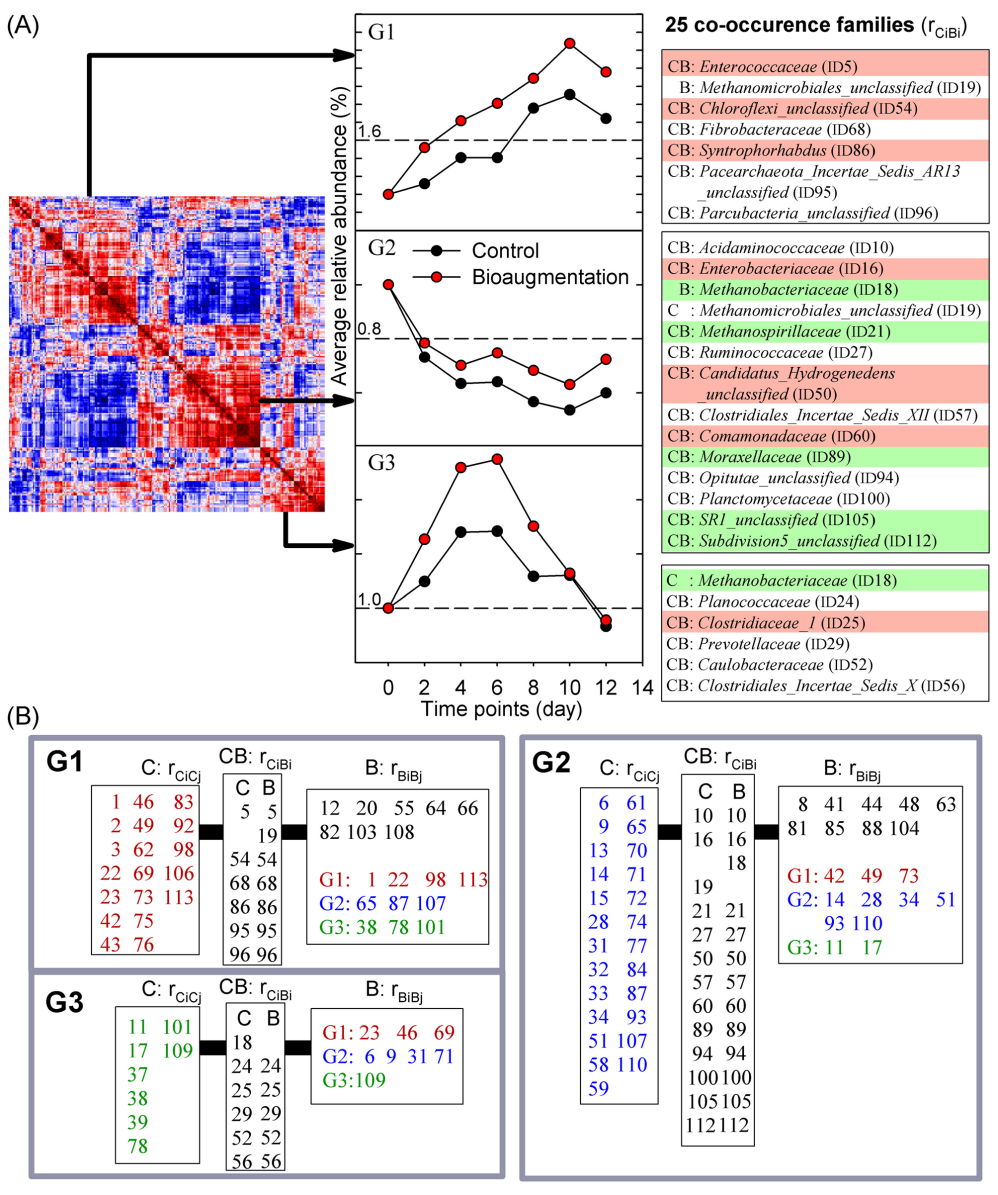
Co-occurrence clusters revealed different co-occurrence abundance patterns. (**A**) 25 microbial families with highly correlated abundance patterns before (control) and after bioaugmentation. (**B**) Three co-occurrence clusters determined using the families in (**A**). The displayed numbers were taxon IDs which could be checked in the Supplementary Table S7. Microbes with differential rises and declines in abundance from Fig. 4A are in red and green. ${\text{r}}_{{{\text{C}}_{{\text{i}}} {\text{B}}_{{\text{i}}} }}$ captured conserved abundance patterns before and after bioaugmentation. Based on the correlated microbes with ${\text{r}}_{{{\text{C}}_{{\text{i}}} {\text{B}}_{{\text{i}}} }}$, microbes with similar abundance pattern, ${\text{r}}_{{{\text{C}}_{{\text{i}}} {\text{C}}_{{\text{j}}} }}$ or ${\text{r}}_{{{\text{B}}_{{\text{i}}} {\text{B}}_{{\text{j}}} }}$, were identified under control or bioaugmentation.

To understand which microbes were influenced by bioaugmentation, three abundance features—abundance level (H/L/R/nR; Fig. 3A), differential abundance status (UP/DN; Fig. 4A), and correlated abundance patterns (also called co-occurrence patterns, ${\text{r}}_{{{\text{C}}_{{\text{i}}} {\text{B}}_{{\text{i}}} }}$; Fig. 5A)—were measured to determine their associations under bioaugmentation pressures. Thirty-three families with significantly differential abundances were identified as indicators of the subtle impact of adding *S. acidaminiphila* on the entire microecosystem, 20 of which increased in abundance and 13 decreased (Fig. 4A). Of these 33 families, eight demonstrated an overall dominance: *Lachnospiraceae*, *Porphyromonadaceae*, *Clostridiales_unclassified*, *Firmicutes_unclassified*, *Bacteria_unclassified*, *Bacteroidales_unclassified*, *Syntrophaceae*, and *Candidatus_Cloacamonas_unclassified*. Apart from *Syntrophaceae* and *Candidatus_Cloacamonas_unclassified*, these dominant microbes increased in abundance after bioaugmentation, but these changes did not appear to be correlated at all before and after bioaugmentation (not in Fig. 5A). Concerning the correlated abundance pattern, 12 highly correlated families were much less abundant (marked with yellow in Fig. 4A).

Therefore, a subtle perturbation could interfere with microbial communities across these three abundance features. Here, we used a contingency table to decipher the intertwined relationship among them (Supplementary Fig. S3). The separation of microbial families based on microbial abundance level was significantly associated with that by differential abundance (p = 1.55 $\times$ 10^–3^) or correlated abundance pattern (p = 2.0 $\times$ 10^–3^), but microbes partitioned by abundance correlation were not significantly associated with those partitioned by differentially abundant attributes (p = 0.165) (Supplementary Fig. S3).

The abundance profiles of *S. acidaminiphila* and methanogens were observed to examine the effects of the subtle perturbation on the entire microbiome. The invariant abundance level of *Xanthomonadaceae* (Fig. 4B), which *S. acidaminiphila* belongs to, suggested that anthropogenic microbial interference only disturbed the microecosystem at a small scale, but it could still temporarily improve biogas and methane yields (Fig. 2 and Supplementary Fig. S2) after bioaugmentation. However, this change in biogas and methane levels during the anaerobic digestion process was not influenced by the prevalence of the archaeal community (Fig. 4C) or methanogens (Table 1), which are known to produce methane from substrates such as H_2_, acetate, methanol, and methylamine. The archaeal community contained methanogenic families and four other unclassified families with a similar or lower abundance after *S. acidaminiphila* was added (Table 1). Therefore, the improvement in biogas and methane production after bioaugmentation may have been caused by bacterial communities (Supplementary Results 3) rather than well-known archaeal methanogens.

**Table 1:** Microbial abundance of archaeal families with and without bioaugmentation by adding *S. acidaminiphila*.

Archaeal families	C	B	*p* value
ID20: *Methanotrichaceae* (A)	15.01 ± 0.42	15.41 ± 0.63	0.331
ID17*:Methanosarcinaceae* (A/H/M1/M2)	0.03 ± 0.01	0.01 ± 0.00	0.088
ID21: *Methanospirillaceae* (H)	0.70 ± 0.56	0.56 ± 0.06	< 0.001*
ID13: *Methanomicrobiaceae* (H)	0.15 ± 0.02	0.14 ± 0.02	0.328
ID18: *Methanobacteriaceae* (H/M1/M2)	0.90 ± 0.06	0.59 ± 0.03	< 0.001*
ID15: *Methanomassiliicoccaceae* (M1)	0.14 ± 0.02	0.11 ± 0.01	0.076
ID39: *Archaea_unclassified*	1.10 ± 0.07	1.05 ± 0.05	0.145
ID67: *Euryarchaeota_unclassified*	0.68 ± 0.04	0.51 ± 0.04	0.014*
ID19: *Methanomicrobiales_unclassified*	0.31 ± 0.03	0.34 ± 0.02	0.253
ID95: *Pacearchaeota_Incertae_Sedis* *_AR13_unclassified*	0.32 ± 0.08	0.30 ± 0.07	0.246

Relative abundance from different time-series samples before and after bioaugmentation are represented as the average and standard error and were tested by Paired-samples Student’s t test (H_0_: U_C_ = U_B_). Acetoclastic, hydrogenotrophic, and methylotrophic methanogens were defined by KEGG modules and are denoted A (M00357: Acetate CH_4_), H (M00567: CO_2_ CH_4_), M1 (M00356: Methanol CH_4_), and M2 (M00563: Methylamine CH_4_).

### Subtle perturbation and co-occurrence patterns

Microbes with co-occurrence patterns were known to be driven by metabolic interactions and competition for resources^26^, and had the potential to capture crucial community characteristics that might not be discovered in microbial diversity or abundance-based analysis^27,28^. Based on 25 highly correlated microbial families (${\text{r}}_{{{\text{C}}_{{\text{i}}} {\text{B}}_{{\text{i}}} }}$) with peculiar co-occurrence (ascent, descent, and convex) abundance patterns (Fig. 5A and Supplementary Fig. S4), three microbial clusters (G1, G2, and G3) were established to capture different subsets of microbial families using ${\text{r}}_{{{\text{C}}_{{\text{i}}} {\text{C}}_{{\text{j}}} }}$ (and ${\text{r}}_{{{\text{B}}_{{\text{i}}} {\text{B}}_{{\text{j}}} }}$) before (and after) bioaugmentation (Fig. 5B and Supplementary Fig. S4). After *S. acidaminiphila* was added, the microbial families with ${\text{r}}_{{{\text{B}}_{{\text{i}}} {\text{B}}_{{\text{j}}} }}$ captured by 25 microbes with co-occurrence (${\text{r}}_{{{\text{C}}_{{\text{i}}} {\text{B}}_{{\text{i}}} }}$) patterns were different to those with ${\text{r}}_{{{\text{C}}_{{\text{i}}} {\text{C}}_{{\text{j}}} }}$ without bioaugmentation. For the ascent abundance pattern, *Porphyromonadaceae* (ID1), *Pseudomonadaceae* (ID22), *Peptococcaceae_2* (ID98), and *Sunxiuqinia* (ID113) were preserved in the G1 cluster, but were attached by different microbes with ${\text{r}}_{{{\text{C}}_{{\text{i}}} {\text{B}}_{{\text{i}}} }}$ (Fig. 5B and Supplementary Fig. S4). The G2 cluster contained microbes with a coherent descent abundance pattern, such as *Corynebacteriaceae* (ID14), *Lachnospiraceae* (ID28), *Actinomycetaceae* (ID34), *Opitutaceae* (ID93), *Streptococcaceae* (ID110), and *Candidatus_Saccharibacteria_unclassified* (ID51), of which only *Lachnospiraceae* showed a differential rise in abundance (Fig. 4). As the only member with a preserved convex abundance pattern in the G3 cluster, *Xanthomonadaceae* (ID109), the family of *S. acidaminiphila*, was linked by *Clostridiaceae_1* (ID25) before bioaugmentation and by *Prevotellaceae* (ID29) after. Although *Xanthomonadaceae* was rarely associated with methane production in previous studies, *Clostridiaceae_1* and *Prevotellaceae* are well-known bacterial families that are involved in hydrolysis, acidogenesis, acetogenesis, and methanogenesis. Furthermore, *S. acidaminiphila* seemed to act as a driver that gathered more core microbes (i.e. H, L and R in Supplementary Fig. S4) to have co-occurrence abundance patterns and more co-occurrence links after bioaugmentation in the G1 and G3 clusters (Supplementary Fig. S4); this might be an innate mechanism to retain special regulations or enhance the production of biogas and methane in the microbial ecosystem.

### Subtle perturbation and microbial network

**Figure 6:**
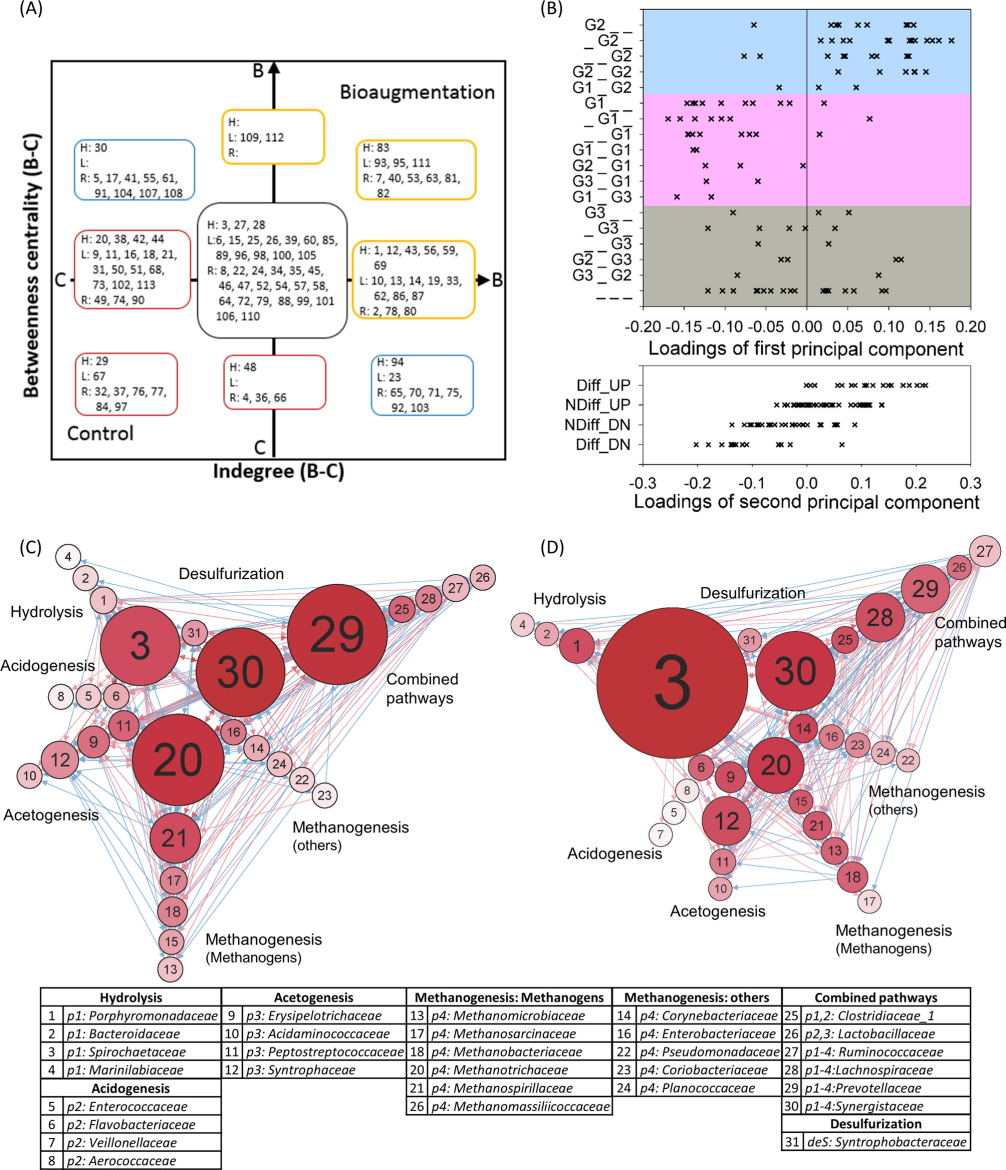
Microbial networks were helpful for detecting the differences with and without adding *S. acidaminiphila*. (**A**) A systematic visualization comparing two microbial networks based on betweenness and indegree centrality in the control and bioaugmentation groups. Microbial IDs in yellow (or red) boxes conveyed larger indegree and betweenness centralities and were more critical for the bioaugmentation (or control) group. Families framed in the blue box showed their superiority for one centrality, indegree or betweenness, and were considered to be ambiguity for topological niche for a group. (**B**) Co-occurrence clusters (G1, G2, or G3) and differential abundance status (Diff_UP/ NDiff_UP/ NDiff_DN/ Diff_DN) explained the variance in the first and second principal components. The family belonging to which co-occurrence clusters before (${\text{r}}_{{{\text{C}}_{{\text{i}}} {\text{C}}_{{\text{j}}} }}$, Gx) and after (${\text{r}}_{{{\text{B}}_{{\text{i}}} {\text{B}}_{{\text{j}}} }}$, Gz) bioaugmentation was denoted as GxGyGz (x, y, or z = 1, 2, 3) where Gy meant microbes with ${\text{r}}_{{{\text{C}}_{{\text{i}}} {\text{B}}_{{\text{i}}} }}$. Principal component loadings were weights of microbial families on the first (or second) principal component. Two small-scale microbial networks including microbes matched to well-known functions are displayed (**C**) without and (**D**) with the addition of *S. acidaminiphila*. The node size and color indicated betweenness and indegree centrality (see Materials and Methods). Diff_UP or Diff_DN indicates microbes with differential abundances that rose or declined, respectively (Fig. 4). NDiff_UP or NDiff_DN indicates microbes with non-differential abundances that rose or declined after adding species (Supplementary Table S12).

The microbial families with highly correlated abundance patterns,${\text{ r}}_{{{\text{C}}_{{\text{i}}} {\text{B}}_{{\text{i}}} }}$ (Fig. 5A), between the control and bioaugmentation condition, and these guided the microsystem into three co-occurrence clusters (Fig. 5B), ${\text{r}}_{{{\text{B}}_{{\text{i}}} {\text{B}}_{{\text{j}}} }}$ and ${\text{r}}_{{{\text{C}}_{{\text{i}}} {\text{C}}_{{\text{j}}} }}$, and turned more core families, denoted as H, L and R in Supplementary Fig. S4, together in a co-occurrence manner. These microbial families with correlated abundance patterns (${\text{r}}_{{{\text{C}}_{{\text{i}}} {\text{B}}_{{\text{i}}} }}$) with and without bioaugmentation may play a role in regulating the entire microbiome and reach the goal of improving biogas and methane production. To decipher the regulatory roles of these 25 microbes with co-occurrence patterns (${\text{r}}_{{{\text{C}}_{{\text{i}}} {\text{B}}_{{\text{i}}} }}$), microbial networks, network topologies, and network motifs from the control and bioaugmentation were used to discern any regulatory patterns in the microbial ecosystem (Fig. 6).

First, two microbial networks with and without bioaugmentation were constructed from time-series samples. Then, the difference between the microbial networks was measured in a systematic way using two topological indices, betweenness, and indegree centrality under different levels of interactive strengths. Microbial families that had higher betweenness and indegree centrality with (or without) bioaugmentation were shown at first (or third) quadrant in Fig. 6A. For the negative control, 12 families specific to conditions with or without bioaugmentation (Fig. 3A) were assembled into networks and their definite topological niche was shown (Fig. 6A). Although microbial families with high abundance levels, abundance differential statuses, and correlated abundance patterns were frequently considered as critical for comparison, they were not always defined as important in an intertwined microsystem (Fig. 6A). For example, two correlated families, *Clostridiaceae_1* (ID25) and *Comamonadaceae* (ID60), had higher abundance levels after bioaugmentation, but showed no topological difference after *S. acidaminiphila* was added (Fig. 6A). However, *Methanomicrobiaceae* (ID 13) had no specific abundance properties, but showed topological niches under the pressure of bioaugmentation.

Then, 3-node and 4-node network motifs were analyzed for each microbial network to determine the regulatory patterns from microbial networks. The strongest microbial interactions (top 500 to 2000) were selected to obtain significant 3-node motif types (Supplementary Table S9). For these 3-node motifs, M3-36 and M3-78, i.e. 3-node motif with motif type ID36 (or ID78), were specific to the microbial network with bioaugmentation. M3-36 was a convergence pattern of microbial relations and M3-78 possessed the bidirectional patterns, convergence and divergence, for microbial interactions. The added species stimulated communication behaviors between microbes. The microsystem could induce or repress a microbial member more quickly with M3-36, and the inferences of an essential microbial family could enlarge signals instantly using M3-78. In addition, M3-74 and M3-98 were important motifs for both microbial networks with and without bioaugmentation. M3-74 was a cascade motif that triggered sequential repressions or activations^29^. The M3-98 motif was a feedback loop that occurs when outputs of a system amplifies or inhibits the system to generate sustained oscillations for biological rhythms^30^.

**Figure 7:**
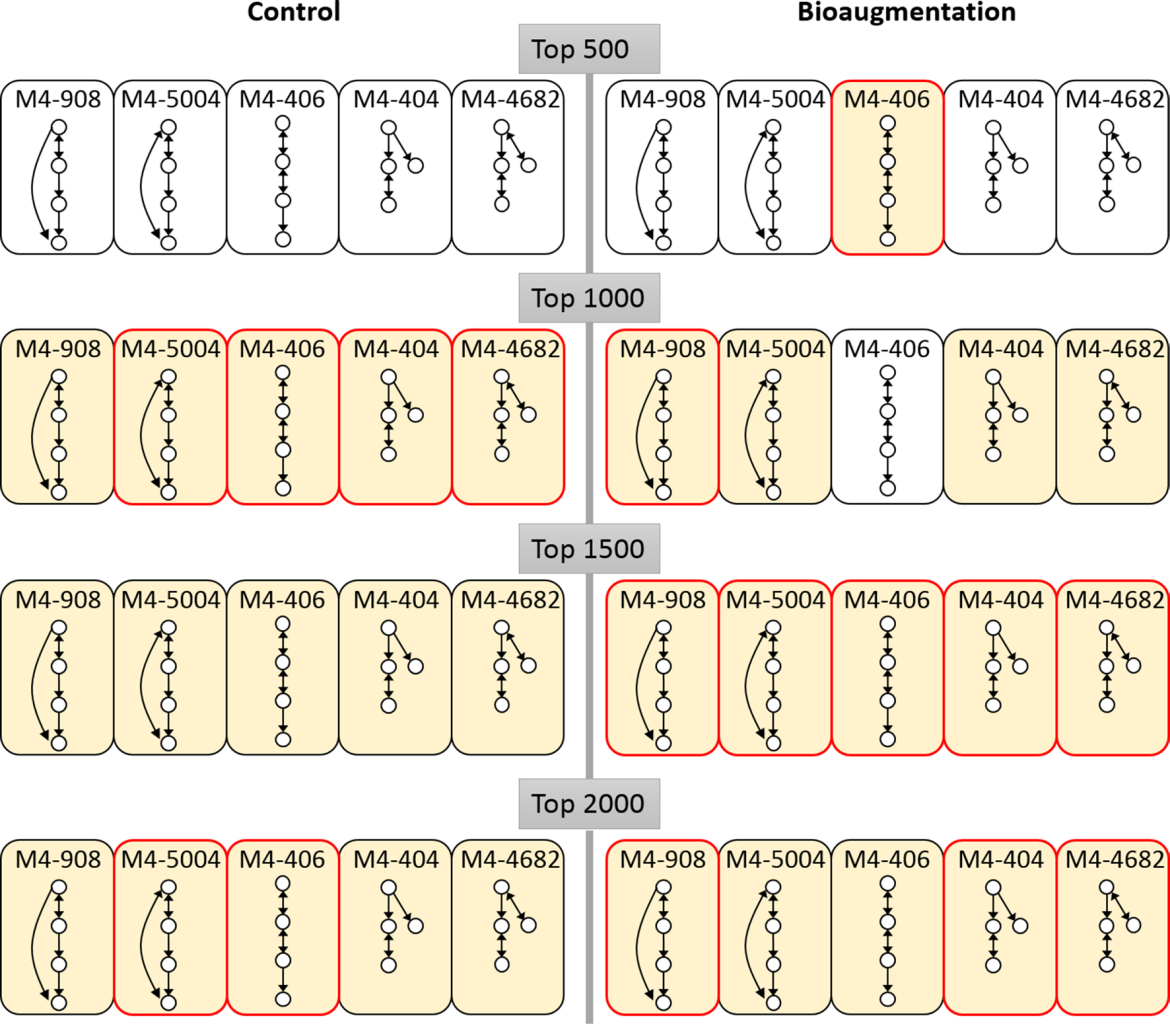
The niche flow of significant 4-node motifs in two microbial networks. Significant 4-node motifs identified by mfinder^65^ under different interaction strengths (top 500 to 2000) are in yellow and insignificant ones are in white. Motifs with higher occupancies in a microbial network (Supplementary Table S11) are framed in red.

To understand the significance and conservation of 3-node motifs in microbial networks, significant 4-node motifs (Supplementary Table S10) were further identified to determine whether or not these 3-node motifs reoccurred. Five 4-node motifs—M4-404, M4-406, M4-908, M4-4682, and M4-5004—were simultaneously defined as significant among microbial networks before and after bioaugmentation (green region in Supplementary Table S10). All three 3-node motifs—M3-36, M3-74, and M3-78—were portions of these 4-node motifs (not feedback motif M3-98). For example, M3-74 was a part of M4-404. The frequencies of these five consistent 4-node motifs in the two microbial networks are listed in Supplementary Table S11 and visualized in Fig. 7. Adding species induced stronger microbial interactions conveying a long chain communication by M4-406 and M4-908, and weaker interactions conveying additional support by a short chain cascade (M4-404 and M4-4682). However, without the help of additive species, the strong interactions were contributed by a mixture of long chain (M4-5004 and M4-406) and 3-node cascade (M4-404 and M4-4682) motif niches, and weaker interactions only preserved the long chain motifs (Fig. 7). The comparison of niche flow among the 4-node motifs revealed potential benefits of adding species, as the niche motifs under different interactive strength levels from M4-406 (top 500), M4-908 (top 1000) to M4-908, M4-404 and M4-4682 (top 2000) formed a hierarchical regulatory structure to function in the ecosystem in a parallel way.

Then, seven and fourteen 4-node motifs specific to microbial networks with and without bioaugmentation were determined, respectively (Supplementary Table S10). Under the condition of adding *S. acidaminiphila*, a quite different group of significant 4-node motifs, especially looped motifs (orange regions in Supplementary Table S10), were identified and seemingly featured in the M3-98 feedback motif. For theses 4-node motifs with feedback loops, higher frequencies of co-occurrence microbial families (${\text{r}}_{{{\text{C}}_{{\text{i}}} {\text{B}}_{{\text{i}}} }}$) (Fig. 5A) were observed after bioaugmentation (Supplementary Fig. S5). When the 500 strongest interactions were selected, seven out of ten 4-node looped motifs had a higher proportion of M3-98 feedback motifs with correlated microbes after bioaugmentation (Supplementary Fig. S5A). For instance, 29.27% of the M4-4418 motif contained the M3-98 feedback motif with correlated microbes (Supplementary Fig. S5A) when the top 500 strong interactions were kept. Similarly, the microbial network without bioaugmentation produced many 4-node loop motifs, such as M4-6874 and M4-13150, which were also contributed to by microbes with co-occurrence abundance patterns (Supplementary Fig. S5A). When the 2000 strongest interactions were used (Supplementary Fig. S5B), a similar association between co-occurrence microbes and the M3-98 feedback loop among 4-node motifs was found. Six 4-node motifs—M4-330, M4-4418, M4-4998, M4-5062, M4-6870, and M4-13142—had more correlated 3-node M3-98 feedback patterns after adding *S. acidaminiphila* when the 2000 strongest interactions were used (Supplementary Fig. S5). M4-6874 still showed a higher percentage of the correlated M3-98 motif without bioaugmentation.

Three abundance features and co-occurrence clustering types were matched with principal component loadings to provide a substantial link between abundance attributes and principal component loadings and decipher the potential drivers separating microbial communities (Figs. 3B, 6B). The main force driving the first and second principal component was derived from discriminating G1 and G2 co-occurrence clusters and four differentially abundant categories, respectively (Fig. 6B and Supplementary Tables S12). These findings led us to understand the importance of recognizing co-occurrence abundance patterns in microbiome research (Fig. 6B), as they might play critical regulatory roles in microbial ecosystems.

To determine the metabolic functional changes between two microbial networks with and without the additional species, two small-scale microbial networks—including families with well-known methane-related metabolic pathways, hydrolysis, acidogenesis, acetogenesis, methanogenesis, and desulfurization, based on our previous study^31^—were constructed (Fig. 6C,D) to examine changes in the topological niche among the five methane-related pathways under bioaugmentation pressure. More interactions of *Porphyromonadaceae* (ID1) and *Bacteroidaceae* (ID2) increased hydrolysis. This was consistent with the observation that adding *S. acidaminiphila* increased chitin and cellulose degradation (Supplementary Results 3). Apart from *Veillonellaceae* (ID7), all microbes that could undergo acidogenesis contributed equally to microbial networks (Fig. 6A,C,D). Then, *Syntrophaceae* (ID12) and *Acidaminococcaceae* (ID10) showed a topological niche after adding *S. acidaminiphila*, but another two were more important without bioaugmentation in the process of acetogenesis (Fig. 6A). Methanogens did not show increased abundance (Table 1), but all except for *Methanomicrobiaceae* (ID13) showed a slight topological niche. Only one syntrophic methanogen, *Corynebacteriaceae* (ID14), showed a significant interactive topology (Fig. 6A). The ambiguous topological niche of *Synergistaceae* (ID30) revealed a larger betweenness centrality after the subtle perturbation and a larger indegree centrality without any perturbation (Fig. 6A,C,D). The metabolic improvement by multifunctional *Clostridiaceae_1* (ID25) and *Lachnospiraceae* (ID28) might generate stronger impacts than that by *Prevotellaceae* (ID29)*.*

These results indicated that integrating intertwined relationships among microbes into a microbial ecosystem helps detect and decipher changes in the microbiome in response to a subtle disturbance. Co-occurrence abundance patterns are a common way to investigate the microbial interactions. In this study, the regulatory role of correlated microbial families (Fig. 5) was further established. Microbes kept special abundance patterns to fine-tune the major metabolic functions and modified the interactive relationships to form hierarchical niche motifs that could respond to the perturbation in a more sensitive way. Therefore, we provided a systematic decomposition of the entire microbial ecosystem after a subtle disturbance by adding *S. acidaminiphila* at a very different aspect.

## Discussion

Microbial diversity is a common bioindicator of ecosystem functions^32^, but it is not sensitive enough to detect chronic and subtle environmental perturbations^5,9^. Detecting or monitoring gradual changes are important for preventing global changes, ecological disturbances, and human-induced pollution from worsening. Instead of biodiversity, non-random patterns in microbial species co-occurrence and associated metrics are being integrated to amplify the differences that subtle perturbation makes^22,33^, e.g. a temperature increase of 1 °C for 5 years^20^, soil contamination with mercury for several decades^17^, and annual litter decomposition^18^. Our analysis provides a framework for studying microbial communities, co-occurrence, and networks under subtle anthropogenic perturbation by adding *S. acidaminiphila*.

In this study, a network based approach was first proposed to design and measure a microbial ecosystem after a subtle perturbation. Based on network topologies, predicted key species were added to a mesophilic anaerobic digestion microsystem to slightly interfere with the priority effect from the species that arrived first in the communities^24,34^. The addition of *S. acidaminiphila* yielded increased biogas and methane production; this represents the transitory disturbance to the microbial ecology, and the invariant microbial species represent a protective mechanism in this microsystem that prevents the colonization and overgrowth of new bacterial species. Therefore, this study takes a step toward designing a novel laboratory-scale that differentiates microbial topological niches in response to subtle perturbation pressure. This whole new way of artificially disturbing a microbial ecosystem at a small scale provides a chance for researchers to observe and investigate changes in microbial communities in response to gradual and subtle perturbation. In addition, the generalization of such laboratory-scale experimental findings to real environmental changes might help monitor and sound the alarm about subtle changes from chronic atmospheric pollution^20^, agricultural practices^18^, and metallic contaminants in the soil^17^ at an early stage, which can then be countered with ecosystem management strategies.

Although most taxa were maintained after *S. acidaminiphila* was added, changes in abundance level, differential abundance status, correlated abundance pattern (${\text{r}}_{{{\text{C}}_{{\text{i}}} {\text{B}}_{{\text{i}}} }}$), network motif, and microbial network were detected. For three abundance attributes, differential abundance status and correlated abundance did not associate with each other, but each was significantly associated with abundance level. The conventional way to decipher microbial communities with high abundance levels is not capable of adequately explaining intertwined microbial relationships. However, co-occurrence patterns and network topology results underscore the importance of recognizing regulatory interactive behaviors between microbes. In three co-occurrence clusters, seven correlated microbes (${\text{r}}_{{{\text{C}}_{{\text{i}}} {\text{B}}_{{\text{i}}} }}$)—*Enterococcaceae* (ID5), *Acidaminococcaceae* (ID10), *Enterobacteriaceae* (ID16), *Fibrobacteraceae* (ID68), *Clostridiales_Incertae_Sedis_XI* (ID56), *Syntrophorhabdus* (ID86), and *Methanomicrobiales_unclassified* (ID19)—were linked with microbes containing the KEGG (Kyoto Encyclopedia of Genes and Genomes) reactions R10204 and R09339 (Supplementary Results 3)^35^.

*Syntrophorhabdus aromaticivorans* (family *Syntrophorhabdus* (ID86)) was the first cultured anaerobe found to be responsible for degrading phenol into acetate and methane in a syntrophic relationship with hydrogenotrophic methanogens^36^. The methane generating potential of *Syntrophorhabdus* (ID86) was ignored in KEGG reactions; however, the co-occurrence patterns between *Syntrophorhabdus* (ID86) and microbes with R10204 and R09339—*Pseudomonadaceae* (ID22), *Desulfuromonadaceae* (ID64), and *Dietziaceae* (ID65)—addressed the underlying importance of generating methane, which was proven in a literature survey^36^. Furthermore, *Syntrophorhabdus* (ID86) increased in abundance with bioaugmentation but not consistently across all samples. *Syntrophorhabdus* (ID86) coupled with *Methanomicrobiales_unclassified* (ID19), *Acidaminococcaceae* (ID10), and *Clostridiales_Incertae_Sedis_XI* (ID56) were also shown to have critical roles in the network topology after *S. acidaminiphila* was added, whereas *Methanomicrobiales_unclassified* (ID19) might include uncultured hydrogenotrophic methanogens that are syntrophic with *Syntrophorhabdus* (ID86). Acetogenesis of *Acidaminococcaceae* (ID10) could definitely raise the efficacy of biogas and methane yields. Besides, some gram-negative short rods in *Escherichia* and *Enterobacter*^37^ (family *Enterobacteriaceae* (ID16)) could enlarge the formation of methylmercury and indirectly improve methane production, even in the presence of oxygen. Concerning the daily feeding of swine manure to the mesophilic anaerobic digesters, it was inevitable that some oxygen entered the microsystem, thus allowing for some bacterial methanogenesis.

These improvements mentioned above might be triggered by adding *S. acidaminiphila*, an aerobe that was first isolated from a lab-scale upflow anaerobic sludge blanket reactor treating petrochemical wastewater^38^. *S. acidaminiphila* has multiple carbon sources, including acetate, crotonate, fumarate, DL-Lactate, pyruvate, and succinate. Furthermore, it can degrade N-Acetylglucosamine^38^—the monomeric unit of the polymer chitin and a major component of the cell walls of most fungi and bacteria—and be resistant to various antimicrobial agents^39^. Consistent with the potential metabolic functions of the family of *Xanthomonadaceae* based on KEGG reactions, *Xanthomonadaceae* conveyed the ability to degrade cellulose (R11307) and chitin (R01206 and R02334). The degradation of N-Acetylglucosamine implies that *S. acidaminiphila* in anaerobic digesters increases fiber digestion and the ability to break down or reuse dead microbes.

Bacteria-oriented methanogenesis and carbon degradation explain why biogas and methane yields increased in a low-abundance methanogenic population after *S. acidaminiphila* was added. In addition, the microbial networks suggest a transition from methanogens that consume a combination of acetate, hydrogen, and methanol (*Methanotrichaceae, Methanospirillaceae,* and *Methanobacteriac*eae) to purely hydrogenotrophic types (*Methanomicrobiaceae* and *Methanomicrobiales_unclassified*). Although the concentration of organic acids could not be detected in this study, it is possible that the added *S. acidaminiphila* rapidly exhausted most of the acetate at anaerobic digesters and created a hydrogen-abundant environment for hydrogenotrophic methanogens. However, more rigorous and extensive laboratory experiments are necessary to support this proposition and make any definitive claims along these lines.

Co-occurrence and model-based microbial networks are two popular approaches based on different rationales to decipher dynamic microbial ecosystems^15,40–43^. The biggest difference between the two prediction strategies is whether the interaction direction can be inferred. Commonly used methods that use co-occurrence networks to infer microbial interactions are based on non-directional association measurements, such as Pearson and Spearman correlation^17,19,44^, Bray–Curtis distance^45^, or covariance estimation^46^. Model-based networks can follow certain regression models, such as Bayesian statistics, Lotka-Volterra, and a variety of sparse regressions^40,47–49^. However, this is the first study to combine co-occurrence and model-based microbial networks (e.g. Lotka-Volterra), and it indicates the regulatory roles that co-occurrence microbes might play in a dynamic microbial network. Although the addition of *S. acidaminiphila* to anaerobic digesters did not directly interfere with microbial community diversities, it did change the microbial ecosystem by enhancing network motifs and motivating bacteria-mediated methanogenesis by feedback loop and cascade signaling motifs.

Consistent with previous studies^50,51^, feedback loop regulation had a great impact on cell growth and microbial biofuel production, where the toxic effects of biofuels for cell growth could be mitigated by expressing efflux pumps to export biofuels from the microbes. Furthermore, the overall performance of biofuel production depended on a cascade process, including the efficient pretreatment of influent sludge, more short-chain fatty acids, and higher conductivities in the fermentative liquid^52^. In our study, the stronger microbial interactions highlighted long chain regulatory cascade motifs, M4-406, and parallelly the weaker interactions featured shorter chain cascade motifs, M4-404 and M4-4682, which may have boosted the microbial cascade process by switching to different interactive strengths after bioaugmentation. Our study demonstrates for the first time under subtly perturbed environments that the purpose of hierarchical regulatory motifs launched by co-occurrence members might form a functional module to respond to the dynamic surroundings instantly.

Previous efforts to characterize ecological fitness and adaptation have primarily been conducted based on the response of microbial diversities to some disturbance. In the coming years, dynamic microbial ecological studies will increasingly be applied to detect subtle environmental perturbations. We present a systematic approach for handling time-series microbial communities to detect slight changes in microbial abundance between two populations with and without subtle perturbation. This method can be generalized to dynamic experiments in a wide variety of fields and provides a predictive direction and landscape for further research and experimental designs. In order to obtain more reliable and objective support, future research should search for evidence of these regulatory network motifs under a microbial ecological process and use them to decipher intertwined relations among microbes.

## Materials and methods

### Bench-top anaerobic digesters

A set of ten 5-L bench-top continuous stirred tank reactors (CSTRs) with a working volume of 3 L was set up for this study^53^. Two anaerobic digesters—one for the control and another one for the bioaugmentation treatment—were operated in May, 2017. To confirm the effect of adding species on biogas and methane yields, eight anaerobic digesters—four each for the control and bioaugmentation treatments—were used to reproduce the process in August, 2017. The CSTR design can completely homogenize the digester content and collect samples in a straightforward manner. The primary feedstock for these digesters was pig manure collected from pig farms in DaXi, ZaoQiao Township, Miaoli County, Taiwan (we received approval from the farm owners to collect concentrated swine manures). These anaerobic digesters were operated under mesophilic (37 °C) conditions using a hydraulic retention time (HRT) of 10 days, 8% total solids (TS), and a stirring speed of 60 rpm without pH control. All anaerobic digesters were initiated using 1.2 L of inoculum from the anaerobic digesters, which were maintained by Dr. Chu-Yang Chou’s laboratory in the Department of Biomechatronics Engineering, National Taiwan University (Taipei, Taiwan), and 1.8 L of swine manure from the pig farms mentioned above.

### Isolation of pure culture from anaerobic digesters

Four kinds of media were used to isolate aerobic bacteria: LB (Luria–Bertani) (Difico), TSB (tryptic soy broth) (Bacto), NB (nutrient broth) (Difico), and R2A (0.5 g protesost peptone (Bacto), 0.5 g casamino acids (Bacto), 0.5 g dextrose (Bacto), 0.5 g soluble starch (Sigma), 0.3 g potassium phosphate (Sigma), 0.5 g yeast extract (Bacto), 0.05 g magnesium sulfate (Sigma), 0.3 g sodium pyruvate (Sigma). All four were solidified by adding 1.5% agar (Bacto). The media were autoclaved for 20 min at 121 °C. Then, an effluent sludge sample from mesophilic (37 °C) anaerobic digesters underwent serial dilution (10^–1^-10^–7^). 100 µL of the 10^–4^-10^–7^ dilution samples was added to the 900 µL media and spread using the streak-plate method with glass bead cylinders. All media with sludge samples were incubated at 37 °C for 1 to 7 days for single colony to emerge on the new plate and be used for pure culture isolation.

Pure cultures were preserved for the subsequent experiments by enriching in the medium at 37 °C in a horizontal shaker at 200 rpm. When the culture reached the log phase or stationary phase, 0.6 mL was maintained in a 1.5 mL tube with 0.4 mL 80% (W/V) glycerol; the glycerol concentration reached 20% (W/V). Finally, the culture was mixed well and stored at -20 °C or -80 °C.

### Biochemical methane potential (BMP) tests

BMP is a simple and inexpensive bioassay that measures relative biodegradability by monitoring cumulative methane production in an anaerobic digestion system^54^. The conventional BMP test typically takes two weeks when the amount of biogas or methane remains the same. This study, however, adopted a simplified three-day BMP test to quickly examine biogas and methane yields for each selected key microbial candidate. The batch tests were carried out using 1L serum bottles (Schott Glaswerke); 450 mL effluents from mesophilic anaerobic digesters and 50 mL swine manure with 8% TS were used, 1 mL of bacterial cell counts, such as *E. miricol*, *S. acidaminiphila*, *B. denitrificans*, and *E. coli*, ranging from 10^3^ to 10^8^ CFU/mL were cultured in aerobic media and added into BMP serum bottles at the first day, flushed with nitrogen at the beginning of the assay, and then placed in a thermostatic water bath at a mesophilic temperature (37 °C). All bottles were connected to a water trap and a gas bag to collect biogas. In BMP test, the key species was only added into the anaerobic digestion system at the beginning.

### Bioaugmentation on CSTR

After the steady state was achieved—chemical oxygen demand (COD) became constant or the production of biogas or methane became invariant—1 mL of a bioaugmented species, *S. acidaminiphila* (7.2 $\times$ 10^9^ CFU/mL), was added to five reactors daily, and another five reactors were maintained as before. After 24-h operation, all manure effluents (300 mL) were collected daily for all anaerobic digesters operated in May and Aug 2017 and kept frozen in a -20 °C freezer and thawed before use. To avoid air leakage into the digester, a peristaltic pump (Masterflex Model No. 7553–80, Cole-Parmer Instrument Co., IL., USA) was connected during the feeding and sampling processes. The pH, TS, and COD (colorimetric method) of the influent and effluent samples were analyzed according to standard methods^55^. Total gas or biogas production was quantified by a wet test gas meter (W-NK-0.5, Shinagawa Co., Tokyo, Japan). The methane composition (CH_4_%) was measured by a gas chromatograph (GC-8700 T, China Chromatography Co., Taiwan) equipped with a thermal conductivity detector (GC-TCD) and a Porapak Q column with helium as the carrier gas, and calibrated with a gas standard consisting of 100% methane. Concerning the cost of 16S rRNA sequencing experiments, time-series effluent samples from the most productive one of five bioaugmented (or control) anaerobic digesters were further processed for DNA extraction, PCR amplification and 16S rRNA amplicon sequencing.

### DNA extraction

To isolate bacterial and archaeal DNA from collected samples, all DNA extractions were performed with the PowerSoil DNA Isolation Kit (Mo Bio Laboratories, USA) according to the manufacturer’s instructions. The quality of DNA extracts was checked by a Nanodrop-1000 Spectrophotometer (Thermo Scientific, Wilmington, DE, USA). The amount of DNA was determined using a Quant-iT dsDNA HS assay kit and a Qubit fluorometer (Invitrogen, Life Technologies, Carlsbad, CA., USA). All above-mentioned procedures were performed in a laminar flow cabinet to avoid contamination.

### PCR amplification and 16S rRNA sequencing

Purified DNA extracts were amplified using the primers of a modified 341F (CCTAYGGGRBGCASCAG) and a modified 806R (GGACTACNNGGGTATCTAAT)^56^ fused with Illumina overhanging adapters, which amplified a DNA fragment of about 533 bp containing the V3 and V4 hypervariable regions of the 16S rRNA gene^57^. PCR amplification was performed in a 30 µL reaction volume using 2X Phusion Flash High-Fidelity PCR Master Mix (Finnzymes Oy, Finland), with the following incubation conditions: 98 °C for 3 min; followed by 25 cycles of 98 °C for 30 s, 56 °C for 30 s, and 72 °C for 30 s; and a final extension of 72 °C for 5 min. After PCR, the DNA products were confirmed by 2% agarose gel electrophoresis with Tris–acetate-EDTA (TAE) buffer (TOOLS Biotechnology Co., Ltd. Taiwan) and purified by NucleoSpin Gel and PCR Clean-up (Macherey–Nagel GmbH & Co. KG, Düren, Germany). After the quality of all cleaned PCR products was confirmed by the Nanodrop-1000 Spectrophotometer (Thermo Scientific, Wilmington, DE, USA), the purified amplicons were processed using the Illumina standard protocol for 16S rRNA sequencing library preparation, and sequenced by the MiSeq platform with the reagent kit v3.

### Sequence processing

For paired-end sequencing of 16S-rRNA gene amplicons, all sequences in the FASTQ format were merged using FLASH^58^ with standard parameters except for the maximum overlap parameter, which was set to 150. The filtering process of merged reads was manipulated by MOTHUR^59^. Primers and low-quality sequences—fewer than 375 bases long, with homopolymers longer than eight nucleotides, or with a quality score < 30—were removed using trim.seqs. After the trimming step, nonredundant sequences were generated by the unique.seqs command, then clustered by a criterion of a 97% sequence similarity using UPARSE^60^, and chimeric sequences were eliminated with the OTU-picking step. The classify.seqs command classified sequences into different taxonomies based on the RDP classifier with rainset14_032015.rdp and a confidence score threshold of 80% in MOTHUR^59^. Sequences assigned to Chloroplast, Mitochondria, Eukaryota, or an unknown kingdom were discarded. 16S rRNA gene copy number was adjusted on taxon abundance^61^ to generate an OTU or abundance table at different taxonomic level. The abundance profiles were normalized by dividing the minimum number sequence reads of all samples and discarded taxa with sequence reads smaller than one. After the adjustment of abundance profiles, relative abundances were computed for the subsequent analysis. All sequenced samples were deposited in the NCBI Short Read Archive under BioProject PRJNA629428 (SRR11649175—SRR11649188).

### Statistical analysis and classification of microbial members

For each microbial abundance table at the different taxonomic levels, a relative abundance, *x*_*ik*_, was derived from *i* = 1,…,*N* microbial members and *k* = 1,…,*t* time-series samples under a specific condition, e.g. with or without bioaugmentation. The averaged abundance level of a microbial member among all samples was calculated as:

1 $$\overline{x}_{i} = \frac{{\mathop \sum \nolimits_{k = 1}^{t} \left( {x_{ik} } \right)}}{{N(x_{ik} > 0)}}$$

Therefore, a microbial member could be defined as having a high or rare abundance level, while the average of abundance levels was larger than 1% or smaller than 0.1% of the total number of sequences. Microbes with a mean relative abundance from 0.1 to 1% were classified as the low abundance level group. Finally, a core microbial member was defined as a microbe conveying relative abundances among all time-series samples.

A paired-samples Student’s t test was performed for samples collected on the same date for the control and bioaugmentation group to identify differences in microbial abundance. Correlated abundance patterns for microbes were measured using the formulas below, where C and B indicate the control and bioaugmentation, respectively.

2 $${\text{r}}_{{{\text{C}}_{{\text{i}}} {\text{B}}_{{\text{i}}} }} = \frac{{\mathop \sum \nolimits_{k = 1}^{t} \left( {x_{ik}^{C} - \overline{x}_{i}^{C} } \right)\left( {x_{ik}^{B} - \overline{x}_{i}^{B} } \right)}}{{\sqrt {\mathop \sum \nolimits_{k = 1}^{t} \left( {x_{ik}^{C} - \overline{x}_{i}^{C} } \right)^{2} } \sqrt {\mathop \sum \nolimits_{k = 1}^{t} \left( {x_{ik}^{B} - \overline{x}_{i}^{B} } \right)^{2} } }}$$

3 $${\text{r}}_{{{\text{C}}_{{\text{i}}} {\text{C}}_{{\text{j}}} }} = \frac{{\mathop \sum \nolimits_{k = 1}^{t} \left( {x_{ik}^{C} - \overline{x}_{i}^{C} } \right)\left( {x_{jk}^{C} - \overline{x}_{j}^{C} } \right)}}{{\sqrt {\mathop \sum \nolimits_{k = 1}^{t} \left( {x_{ik}^{C} - \overline{x}_{i}^{C} } \right)^{2} } \sqrt {\mathop \sum \nolimits_{k = 1}^{t} \left( {x_{jk}^{C} - \overline{x}_{j}^{C} } \right)^{2} } }}$$

${\text{r}}_{{{\text{C}}_{{\text{i}}} {\text{B}}_{{\text{i}}} }}$ calculated the Pearson correlation for abundance profiles of a microbe before and after bioaugmentation. ${\text{r}}_{{{\text{C}}_{{\text{i}}} {\text{C}}_{{\text{j}}} }}$ or ${\text{r}}_{{{\text{B}}_{{\text{i}}} {\text{B}}_{{\text{j}}} }}$ measured Pearson correlation between two different microbes under the same conditions. The aim of ${\text{r}}_{{{\text{C}}_{{\text{i}}} {\text{B}}_{{\text{i}}} }}$ was to identify microbes with conserved abundance patterns under the control and bioaugmentation treatments. Under the same condition (control or bioaugmentation), ${\text{r}}_{{{\text{C}}_{{\text{i}}} {\text{C}}_{{\text{j}}} }}$ or ${\text{r}}_{{{\text{B}}_{{\text{i}}} {\text{B}}_{{\text{j}}} }}$ was used to capture microbes with similar abundance patterns to microbes with significant ${\text{r}}_{{{\text{C}}_{{\text{i}}} {\text{B}}_{{\text{i}}} }}$. All statistical significance levels in this study were set to 0.05.

To understand how microbes with co-occurrence patterns, ${\text{r}}_{{{\text{C}}_{{\text{i}}} {\text{B}}_{{\text{i}}} }}$, ${\text{r}}_{{{\text{C}}_{{\text{i}}} {\text{C}}_{{\text{j}}} }}$, and ${\text{r}}_{{{\text{B}}_{{\text{i}}} {\text{B}}_{{\text{j}}} }}$ influenced the entire microbiome, co-occurrence clusters were determined using the following steps. First, two abundance profiles from the control and bioaugmentation treatments were combined and the clustering process was performed using the average linkage method with Pearson correlation, which was conducted on the generalized association plots software^62^. Second, the abundance correlations (${\text{r}}_{{{\text{C}}_{{\text{i}}} {\text{C}}_{{\text{j}}} }}$ or ${\text{r}}_{{{\text{B}}_{{\text{i}}} {\text{B}}_{{\text{j}}} }}$) of microbes in the same co-occurrence cluster were tested for statistical significance, and one of the paired microbes should have been identified as having a statistically significant ${\text{r}}_{{{\text{C}}_{{\text{i}}} {\text{B}}_{{\text{i}}} }}$. Microbes with significant correlations in ${\text{r}}_{{{\text{C}}_{{\text{i}}} {\text{C}}_{{\text{j}}} }}$ or ${\text{r}}_{{{\text{B}}_{{\text{i}}} {\text{B}}_{{\text{j}}} }}$ were retained in the cluster. Finally, microbes with significant ${\text{r}}_{{{\text{C}}_{{\text{i}}} {\text{C}}_{{\text{j}}} }}$(or ${\text{r}}_{{{\text{B}}_{{\text{i}}} {\text{B}}_{{\text{j}}} }}$) were classified as having a conserved abundance pattern before (or after) bioaugmentation. The abundance patterns of these microbes should be heavily influenced to one of the microbes with significant ${\text{r}}_{{{\text{C}}_{{\text{i}}} {\text{B}}_{{\text{i}}} }}$.

### Inference in the microbial interaction network

To infer microbial interactions, a simulation prediction process based on the Lotka-Volterra model was performed by MetaMIS^40^. For each condition (control or bioaugmentation), we generated 1000 microbial networks from the microbial abundance profile with N microbial members and T time-series samples. Each microbial network was randomly predicted by 90% of N microbial members. Then, microbial interactions with 90% coherent interactive outcomes among all predicted interactions were considered reliable after proportional tests were performed; they were used in the subsequent analysis and to construct a microbial network.

### Topological niche of microbial networks

Three topological measurements—indegree, betweenness, and eigenvector centralities—were conducted using Gephi software^63^ for each microbial network, in which a node indicated a microbial member *i* and an edge represented the interactive relation between two microbes. Indegree centrality measured how many other microbes influenced a target microbe and was indicated by node color. A larger indegree centrality (darker red) implies that this microbe was influenced by more microbes or interactions. In a microbial network, two microbes must have more than one path. Therefore, betweenness centrality of a microbe is the number of these shortest paths that passed this microbe, and was represented by node size. A larger node indicates a microbe with a larger betweenness centrality. Eigenvector centrality measures the importance of a microbe in a network based on its connections; a microbe was deemed important if it was easy to link it to other influential microbes and it was located in the center of the network.

To systematically identify microbes with a topological niche between two microbial networks (B and C), indegree and betweenness centralities at different interactive strengths were calculated and compared for tow microbial networks.

4 $$Niche_{i}^{Centrality} = \frac{{\mathop \sum \nolimits_{S = top500}^{All interactions} \left( {Centrality_{i,S}^{B} - Centrality_{i,S}^{C} } \right)}}{{max\left\{ {\left[ {Centrality_{i,S}^{B} - Centrality_{i,S}^{C} } \right]_{S = top500}^{All interactions} } \right\}}}$$

Thus, there were two types of $Niche_{i}^{Centrality}$—$Niche_{i}^{Indegree}$ and $Niche_{i}^{Betweenness}$—and it ranged from -1 and 1. If a microbe’s $Niche_{i}^{Centrality}$ was -0.1 to 0.1, it was considered as non-influential topological niche or there was no difference between the control and bioaugmentation treatments. If a microbe’s $Niche_{i}^{Indegree}$ and $Niche_{i}^{Betweenness}$ were > 0.1, it was identified as having a topological niche after bioaugmentation. When both $Niche_{i}^{Indegree}$ and $Niche_{i}^{Betweenness}$ were < -0.1, the microbe was inferred to have a topological niche in the control group. When a microbe had one positive and one negative values of $Niche_{i}^{Indegree}$ and $Niche_{i}^{Betweenness}$, it was considered to be part of an ambiguous topological niche between the control and bioaugmentation treatments.

### Inferring motifs from microbial networks

Network motifs can reflect biological structures, connections, or regulations in a microbial network^64^. In this study, a network motif-detecting software called mfinder^65^ was used to identify significant 3-node and 4-node motifs at four interactive strengths—the 500, 1000, 1500, and 2000 strongest interactions—for two microbial networks. If a significant 4-node motif was repeatedly defined as having three or more than three interactive strengths, it was considered a representative 4-node motif in a microbial network. If a 4-node motif was representative in the control or bioaugmented microbial networks, motif zscores calculated by mfinder were directly used to compare the significance of this motif in that microbial network.

### Assigning metabolic reactions for microbes

Each microbial member was assigned metabolic functions by KEGG reactions^35^. Queried items including “methane,” “cellulose,” “chitin,” etc. were checked for every KEGG reactions among all microbial strains, which were retained if the genus or family of the strain also occurred in our abundance profiles.

## Supplementary information

Supplementary Information.

Supplementary Table S2.

Supplementary Table S7.

Supplementary Table S12.

Supplementary file01_C_Top500_motif3.

Supplementary Information 6. Supplementary file02_C_Top1000_motif3.

Supplementary Information 7. Supplementary file03_C_Top1500_motif3.

Supplementary Information 8. Supplementary file04_C_Top2000_motif3.

Supplementary Information 9. Supplementary file05_B_Top500_motif3.

Supplementary Information 10. Supplementary file06_B_Top1000_motif3.

Supplementary Information 11. Supplementary file07_B_Top1500_motif3.

Supplementary Information 12. Supplementary file08_B_Top2000_motif3.

Supplementary Information 13. Supplementary file09_C_Top500_motif4.

Supplementary Information 14. Supplementary file10_C_Top1000_motif4.

Supplementary Information 15. Supplementary file11_C_Top1500_motif4.

Supplementary Information 16. Supplementary file12_C_Top2000_motif4.

Supplementary Information 17. Supplementary file13_B_Top500_motif4.

Supplementary Information 18. Supplementary file14_B_Top1000_motif4.

Supplementary Information 19. Supplementary file15_B_Top1500_motif4.

Supplementary Information 20. Supplementary file16_B_Top2000_motif4.
