# Supplementary material for: A network-based approach to deciphering a dynamic microbiome’s response to a subtle perturbation
Source: Sci Rep. 2020 Nov 11;10:19530. doi: 10.1038/s41598-020-73920-5 (PMC7659003; doi:10.1038/s41598-020-73920-5)
Supplement: Supplementary file 1 — Supplementary Information. [file 41598_2020_73920_MOESM1_ESM.pdf]

Supplementary Information

A network-based approach to deciphering a dynamic microbiome's response to a subtle perturbation

Grace Tzun-Wen Shaw[1], An-Chi Liu[2,3]*, Chieh-Yin Weng[1], Yi-Chun Chen[3], Cheng-Yu Chen[1], Francis Cheng-Hsuan Weng[1], Daryi Wang[1] and Chu-Yang Chou[2,3]*

[1]Biodiversity Research Center, Academia Sinica, Taipei 115, Taiwan

[2] Bioenergy Research Center, National Taiwan University, Taipei, Taiwan

[3]Department of Biomechatronics Engineering, National Taiwan University, Taipei, Taiwan

* Corresponding author

Email: CYC*: choucy@ntu.edu.tw DW: dywang@gate.sinica.edu.tw
GTWS: tzunwen@gmail.com ACL*: d00631001@ntu.edu.tw
CYW: yweng6434@gate.sinica.edu.tw YCC: eve801220@gmail.com
CYC: initialj1028@gmail.com FCHW: fchweng@gmail.com

Supplementary Results:

Sup. Results 1: Four decisive criteria to identify key species

We hoped to select key microbial candidates which were potentially critical to produce biogas in the mesophilic condition but the low level of abundance might limit their efficacy. So, adding key microbial species into anaerobic digestion system was possible to influence the microbial ecosystem instantly and increase the biogas production temporarily. The selection of supplemental microbial species added was based on a novel network-based approach (Fig. 1) with four decisive criteria proposed by this study. 1) Unlike commonly used bioaugmentation strategies based on well-known bacterial metabolic functions, key microbial families were identified using a comparative process based on topological differences between microbial networks from mesophilic and thermophilic anaerobic digestion systems (Fig. 1)[1]. Microbial families with topological niches in the mesophilic microbial network (Supplementary Table S1 and S2) and low levels of relative abundance (Supplementary Fig. S1) in the mesophilic condition were marked as having the potential to enhance biogas or methane yields by bioaugmentation. 2) Checking the abundance of key microbial candidates at low levels (Supplementary Figure S1) is critical to reaching the goal of using bioaugmented species to amplify metabolic functions, resist the early colonizing community, and temporarily maximize the impact on a stable microecosystem. 3) Key microbial families should be aerobic in this study because they are easier to manipulate and cultivate. 4) Any bacterial species that could be independently isolated from anaerobic digesters in the laboratory and successfully cultivated in the medium could be retained and tested in the subsequent BMP (biochemical methane potential) and CSTR (continuous stirred tank reactors) experiments, which were used to confirm that the subtle perturbation was successful by searching for increased biogas or methane yields. Microbial species that could cause a subtle perturbation in a stable microbial ecosystem were artificially identified following these four steps (Supplementary Table S1 and S2).

Sup. Results 2: Artificial subtle perturbation by four selected species

To simulate an artificial subtle perturbation in a microbial ecosystem, two aerobic species—*Elizabethkingia miricola* (Family: *Flavobacteriaceae*) and *Stenotrophomonas acidaminiphila* (Family: *Xanthomonadaceae*)—with low abundance (Supplementary Fig. S1) and topological differences (Fig.1, Supplementary Table S1 and S2)[1] were isolated for use as bioaugmented factors to enhance biogas and methane production in mesophilic anaerobic digesters. Furthermore, *Brachymonas denitrificans* (Family: *Comamonadaceae*) and *Escherichia coli* (Family: *Enterobacteriaceae*) were selected for the negative control.

A simplified, three-day version of the BMP test was used to quickly assess the effects of bioaugmentation using these four bacterial aerobes on biogas and methane production. Compared to the control group, *E. miricola* improved the mesophilic anaerobic digestion process of swine manure by the second day, and increased biogas and methane productions by 6.63±3.01% ($p=2.8\times10^{-2}$) and 29.07±8.00% ($p=3\times10^{-3}$), respectively. *S. acidaminiphila* addition increased the biogas and methane production by 5.07±2.61% ($p=4.2\times10^{-2}$) and 14.37±3.99% ($p=3\times10^{-3}$), respectively, compared to the control group on the first day. Biogas and methane production increased due to an absolute increase in methane content (CH_4%) of 1.97±0.30% (or 6.38±1.07%) for *S. acidaminiphila* (or *E. miricola*) on the first (or second) day (Supplementary Table S3). On the other hand, *B. denitrificans* and *E. coli* were predicted to lower biogas and methane productions (Supplementary Table S1), and the BMP test found that they did (Supplementary Table S3).

To further appraise the efficacy of bioaugmented species, *S. acidaminiphila* was chosen because of how quickly it increased biogas and methane yields and was tested again in the CSTR reactors with the daily feeding of swine manure; this was carried out for longer than the BMP test and was regarded as a laboratory-scale biogas plant. The bioaugmented CSTR reactors using *S. acidaminiphila* showed a significantly enhanced gas production rate (GPR) from 1.92±0.02 to 2.05±0.02 L/L/day ($p=8.13\times0^{-5}$) and methane production rate (MPR) from 1.24±0.02 to 1.32±0.03 L/L/day ($p=1.72\times10^{-4}$), meaning that it had successfully disrupted the stable microsystem (Fig. 2). To confirm the effect of *S. acidaminiphila* on biogas and methane yields, eight anaerobic digesters—four each for the control and

bioaugmentation treatments—were used to reproduce the process (Supplementary Fig. S2).

The final step to check whether the subtle disturbance was artificially made was to confirm the biodiversity of microbial communities from the mesophilic anaerobic digesters with and without *S. acidaminiphila* bioaugmentation. 16S rRNA sequencing information and taxonomic classification of time-series samples from anaerobic digesters with and without bioaugmentation were shown in Supplementary Table S4 and S5. The similarities in the Shannon index and Chao-1 richness at the family level among all samples suggested that these biodiversity measurements failed to detect the subtle perturbation caused by the bioaugmentation (Supplementary Table S6). Therefore, a subtle perturbation was successfully performed in the laboratory scale anaerobic digestion system based on the topological niche of microbial networks, meaning that this is a novel and prominent method for identifying potential influencers that can slightly disturb a stable microecosystem.

Sup. Results 3: Generating methane via bacterial KEGG reactions

Six methanogens changed in abundance (Table 1), and those with similar (ID13, 15, 17, and 20) or lower (ID18 and 21) abundance levels after *S. acidaminiphila* was added suggest the existence of unknown bacteria that improved biogas or methane yields. A systematic way to screen bacterial members that might generate methane could rely on metabolic reactions from the KEGG database[2]. The KEGG database found that bacteria containing the KEGG reactions R10204 and R09339 are believed to produce methane by metabolizing methylphosphonate[3] and methylmercury[4] with carbon-phosphorus lyase (EC 4.7.1.1) and alkylmercury lyase (EC 4.99.1.2).

R10204: alpha-D-Ribose 1-methylphosphonate 5-phosphate
+ S-Adenosyl-L-methionine + Reduced acceptor <=>
alpha-D-Ribose 1,2-cyclic phosphate 5-phosphate + **Methane**
+ L-Methionine + 5'-Deoxyadenosine + Acceptor

R09339: Methylmercury + H+ <=> **Methane** + Mercury(2+)

Some bacteria use methylphosphonate as a source of phosphorus—an essential element for nucleic acids, carbohydrates, and phospholipids—when there is a lack of phosphate; this includes most gram-negative bacteria[2,3] and species in *Enterobacteriaceae* (ID16), *Pseudomonadaceae* (ID22), *Coriobacteriaceae* (ID23),

Desulfomicrobiaceae (ID63), *Desulfuromonadaceae* (ID64), *Geobacteraceae* (ID72), and *Rhodobacteraceae* (ID103) and those distributed in three co-occurrence clusters (Supplementary Table S6). Other than *Coriobacteriaceae* (ID23), which had low abundance, most of these families were relatively rare. Two rare families, *Desulfomicrobiaceae* (ID63) and *Desulfuromonadaceae* (ID64), increased dramatically in abundance after bioaugmentation. *Desulfomicrobiaceae* (ID63) (Fig. 3(A)), for example, initially could not be measured, but it became detectable after bioaugmentation, suggesting that the addition of *S. acidaminiphila* stimulated *Desulfomicrobiaceae* growth.

Methylmercury is formed from inorganic mercury directly by microbes in certain aquatic environments or indirectly by acute anthropogenic poisoning, e.g. industrial discharge and large scale combustion of fossil fuels or wastes containing inorganic mercury[4,5]. Methylmercury bioaccumulates in aquatic food chains, making fish meal fed to growing pigs the main source of methylmercury from anaerobic digesters. Five families—*Corynebacteriaceae* (ID14), *Dietziaceae* (ID65), *Geobacteraceae* (ID72), *Moraxellaceae* (ID89), and *Sphingomonadaceae* (ID108)—possess the enzyme alkylmercury lyase (Supplementary Table S6). The abundance of these families remained low (Supplementary Fig. S5). *Dietziaceae* (ID65) was the only one that increased differentially in abundance in the G1 co-occurrence cluster after bioaugmentation (Fig. 4 and Supplementary Table S6). These microbial families with the KEGG reactions R10204 and R09339 were connected by seven central correlated microbes: *Enterococcaceae* (ID5), *Acidaminococcaceae* (ID10), *Enterobacteriaceae* (ID16), *Fibrobacteraceae* (ID68), *Clostridiales_Incertae_Sedis_XI* (ID56), *Syntrophorhabdus* (ID86), and *Methanomicrobiales_unclassified* (ID19). Therefore, co-occurrence clusters were used to form a list of microbes that may be involved in methane production, and this is further discussed in the discussion section.

Furthermore, KEGG reactions also suggested that *Xanthomonadaceae* (ID109) have other functions, including the degradation of cellulose (R11307) and chitin (R01206 and R02334). There reactions contained cellulase (EC 3.2.1.4) and chitinase (EC 3.2.1.14) to degrade common feed ingredients for swine. Although the abundance level of *Xanthomonadaceae* (ID109) was similar with and without bioaugmentation, 28 microbial families (data not shown) that could degrade cellulose and chitin were collected in this study and showed significantly different increases in abundances

($p=1.0\times10^{-2}$) under bioaugmentation pressure. Therefore, adding *S. acidaminiphila* transitorily raised the biogas and methane production, possibly due to the enhancement of bacterial methanogenesis and improvement of carbohydrate degradation. This study points to new possibilities for future microbiome research.

Supplementary Tables:

Table S1 Key microbial families identified by topological niche of microbial networks from mesophilic and thermophilic anaerobic digesters[1]. Ind, Bet and Eig indicated indegree, betweenness, and eigenvector centralities respectively under the strongest 1000 microbial interactions from mesophilic and thermophilic microbial networks. Microbial family with larger centrality (Ind, Bet or Eig) implied its more important role in a microbial network. *E. miricol* and *S. acidaminiphila* were expected to enhance biogas production but *B. denitrificans* and *E. coli* weren't.

**Key families (Species)**	**Mesophilic microbial network**			**Thermophilic microbial network**			**Prediction**
	**Ind**	**Bet**	**Eig**	**Ind**	**Bet**	**Eig**	
Flavobacteriaceae (*E. miricol*)	23	4.85	0.79	15	0.30	0.41	Enhance
Xanthomonadaceae (*S. acidaminiphila*)	29	125.6	0.97	0	0	0	Enhance
Comamonadaceae (*B. denitrificans*)	32	143.7	0.99	40	68.6	0.9	may not enhance
Enterobacteriaceae (*E. coli*)	11	0	0.43	7	0	0.23	may not enhance

Table S3 The effect of bioaugmentation on GPR, MPR and CH_4 %. *E. miricol* and *S. acidaminiphila* were selected to potentially enhance the biogas or methane yields. *B. denitrificans* and *E. coli* were predicted to reduce the production of biogas or methane. Samples were tested by Student's-t test with null hypothesis that sample mean is smaller (or larger) than zero. The label of * or # represents that the mean value is statistically significantly larger or smaller than zero and conveys a p value smaller than 0.05.

	**Day 1**	**Day 2**	**Day 3**
E. miricol			
$\frac{GPR(B)-GPR(C)}{GPR(C)}$ (%)	2.17 ± 1.80 (P = 0.130)	6.63 ± 3.01* (P = 0.028)	60.97 ± 3.64* (P < 0.001)
$\frac{MPR(B)-MPR(C)}{MPR(C)}$ (%)	5.53 ± 3.23 (P = 0.060)	29.07 ± 8.00* (P = 0.003)	104.77 ± 15.10* (P < 0.001)
$CH_4(B)-CH_4(C)$ (%)	0.55 ± 0.42 (P = 0.112)	6.38 ± 1.07* (P < 0.001)	9.02 ± 2.01* (P < 0.001)
S. acidaminiphila			
$\frac{GPR(B)-GPR(C)}{GPR(C)}$ (%)	5.07 ± 2.61* (P = 0.042)	2.87 ± 2.84 (P = 0.169)	-0.11 ± 2.18 (P = 0.519)
$\frac{MPR(B)-MPR(C)}{MPR(C)}$ (%)	14.37 ± 3.99* (P = 0.003)	1.36 ± 3.39 (P = 0.349)	-1.26 ± 1.61 (P = 0.772)
$CH_4(B)-CH_4(C)$ (%)	1.97 ± 0.30* (P < 0.001)	-0.88 ± 0.61 (P = 0.84)	-0.58 ± 0.51 (P = 0.856)
B. denitrificans			
$\frac{GPR(B)-GPR(C)}{GPR(C)}$ (%)	-4.11 ± 0.90# (P < 0.001)	-12.80 ± 3.49# (P = 0.003)	-6.77 ± 6.74 (P = 0.171)
$\frac{MPR(B)-MPR(C)}{MPR(C)}$ (%)	-26.59 ± 2.85# (P < 0.001)	-14.96 ± 7.63# (P = 0.041)	-12.18 ± 12.97 (P = 0.186)
$CH_4(B)-CH_4(C)$ (%)	-4.90 ± 0.49# (P < 0.001)	-0.89 ± 1.32 (P = 0.258)	-2.99 ± 2.13 (P = 0.097)
E. coli			
$\frac{GPR(B)-GPR(C)}{GPR(C)}$ (%)	-2.83 ± 1.15# (P = 0.019)	-6.15 ± 2.49# (P = 0.018)	-5.45 ± 3.24 (P = 0.063)
$\frac{MPR(B)-MPR(C)}{MPR(C)}$ (%)	-23.82 ± 5.00# (P < 0.001)	-28.71 ± 6.54# (P < 0.001)	-28.01 ± 6.29# (P < 0.001)
$CH_4(B)-CH_4(C)$ (%)	-4.68 ± 1.03# (P < 0.001)	-8.4 ± 1.52# (P < 0.001)	-6.14 ± 0.93# (P < 0.001)

Table S4 16S rRNA sequence information of time-series samples from mesophilic anaerobic digesters with and without bioaugmentation.

Experimental group	Time (day)	Raw	After sequence preprocessing	After sequence preprocessing and gene copy number correction
Control	0	74,796	54,948	22,048
Control	2	60,185	43,806	17,506
Control	4	70,047	50,468	20,240
Control	6	70,830	52,873	21,304
Control	8	65,599	49,070	19,638
Control	10	54,995	41,328	16,390
Control	12	85,268	63,054	25,020
Bioaugmentation	0	52,535	38,865	15,398
Bioaugmentation	2	83,101	60,829	24,089
Bioaugmentation	4	42,499	29,711	11,669
Bioaugmentation	6	64,512	47,651	18,919
Bioaugmentation	8	63,815	47,551	18,952
Bioaugmentation	10	77,594	58,176	23,363
Bioaugmentation	12	56,194	42,203	16,955

Table S5 Taxonomic classification under different taxonomic levels. The original abundance tables represented the results from the classify.seqs command by MOTHUR[6]. The adjusted abundance tables were preprocessed abundance profiles.

Taxonomic level	$N(OTU_{original})$	$N(OTU_{adjusted})$	% $(OTU_{adjusted})$
Superkingdom	2	2	100.00%
Phylum	31	29	93.55%
Class	55	51	92.73%
Order	84	71	84.52%
Family	144	113	78.47%
Genus	250	167	66.80%

Table S6 Similar microbial biodiversities between control and bioaugmentation group at the taxonomic level of family. Bioaugmentation was performed by adding *S. acidaminiphila* in the CSTR anaerobic digestion system. Shannon index and Chao-1 richness were shown in average and standard deviation. The similar Shannon index (p =0.08) and Chai-1 richness (p=0.18) were tested by paired-samples Student's t test.

**Experimental group**	**Time (day)**	**Shannon index**	**Chao-1 richness**
Control	0	3.19±0.027	97.26±16.78
Control	2	3.11±0.026	91.07±11.73
Control	4	3.10±0.026	90.66±13.50
Control	6	3.13±0.026	94.32±12.10
Control	8	3.10±0.027	88.68±10.82
Control	10	3.10±0.027	89.77±11.34
Control	12	3.09±0.026	86.34±10.66
Bioaugmentation	0	3.08±0.027	94.75±14.04
Bioaugmentation	2	3.09±0.027	90.54±12.08
Bioaugmentation	4	3.13±0.026	95.27±13.04
Bioaugmentation	6	3.10±0.026	92.82±11.28
Bioaugmentation	8	3.09±0.027	91.10±11.59
Bioaugmentation	10	3.08±0.027	92.12±11.30
Bioaugmentation	12	3.07±0.027	88.24±12.05

Table S8 Methanogens and bacteria that could produce methane via different metabolic pathway were listed in three co-occurrence clusters. The notation of L and R indicated core microbes with low or rare abundance, and nR was non-core microbes with rare abundance. Methanogens were denoted as 1. Families involved in KEGG reaction R10204 and R09339 were marked as 2 and 3. Microbes with differential abundance risen and decline stood for UP and DN based on results from Fig. 4(A).

	**C**	**CB**	**B**
**G1**	*Pseudomonadaceae* (ID22^{DN})^{2R}<br>*Coriobacteriaceae* (ID23)^{2L}		*Pseudomonadaceae* (ID22^{DN})^{2R}<br>*Desulfuromonadaceae* (ID64^{UP})^{2R}<br>*Dietziaceae* (ID65^{UP})^{3R}<br>*Methanotrichaceae* (ID20)^{1R}<br>*Rhodobacteraceae* (ID103)^{2R}<br>*Sphingomonadaceae* (ID108)^{3R}
**G2**	*Dietziaceae* (ID65^{UP})^{3R}<br>*Methanomicrobiaceae* (ID13)^{1R}<br>*Methanomassiliicoccaceae* (ID15)^{1R}<br>*Corynebacteriaceae* (ID14)^{3L}<br>*Geobacteraceae* (ID72)^{2,3nR}	*Methanobacteriaceae (*ID18^{DN})^{1R}<br>*Methanospirillaceae* (ID21^{DN})^{1R}<br>*Moraxellaceae* (ID89^{DN})^{3L}<br>*Enterobacteriaceae* (ID16^{UP})^{2L}	*Desulfomicrobiaceae* (ID63^{UP})^{2nR}<br>*Methanosarcinaceae* (ID17)^{1nR}<br>*Corynebacteriaceae* (ID14)^{3L}
**G3**	*Methanosarcinaceae* (ID17)^{1nR}	*Methanobacteriaceae* (ID18^{DN})^{1R}	*Coriobacteriaceae* (ID23)^{2L}

Table S9 Significant 3-node motifs, marked as circles, in two microbial networks under different cutoff of microbial interactive strengths (MIs). M3-XX means node-3 motif with motif type IDXX. Microbes occupy which 3-node or 4-node motifs can be found in Supplementary files 01-16.

Node-3 motif		Control				Bioaugmentation			
Top n MIs		500	1000	1500	2000	500	1000	1500	2000
M3-36	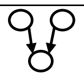						O		O
M3-74	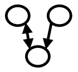	O	O				O		
M3-78	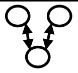					O			
M3-98	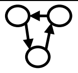			O		O	O	O	O

Table S10 Significant 4-node motifs in two microbial networks. M4-XXX means node-4 motif with motif type IDXXX.

**Common motifs**			
M4-404<br>(M3-74)	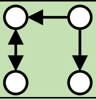	M4-4682<br>(M3-74)	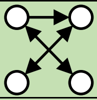
M4-406<br>(M3-78)	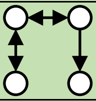	M4-5004<br>(M3-74, 78)	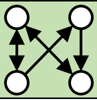
M4-908<br>(M3-36)	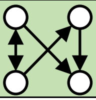		
**Specific motifs in Control group**		**Specific motifs in Bioaugmentation group**	
M4-6874	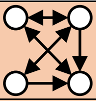	M4-330<br>(M3-98)	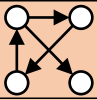
M4-13150	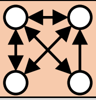	M4-4418<br>(M3-36, 98)	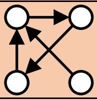
M4-856<br>(M3-36)	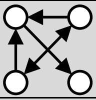	M4-4426<br>(M3-74, 98)	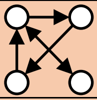
M4-4420<br>(M3-74)	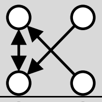	M4-4438<br>(M3-74, 78)	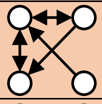
M4-4428<br>(M3-74, 78)	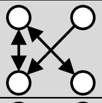	M4-4998<br>(M3-36, 74, 98)	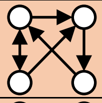
M4-4548<br>(M3-74)	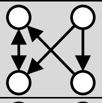	M4-5062<br>(M3-74, 78, 98)	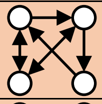
M4-4748<br>(M3-74)	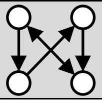	M4-6554<br>(M3-36, 74, 78)	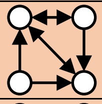
		M4-6598<br>(M3-36, 74, 98)	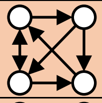
		M4-6870<br>(M3-98)	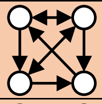
		M4-13142	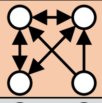
		M4-390	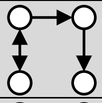
		M4-4370<br>(M3-36, 74)	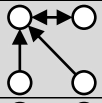
		M4-4812.<br>(M3-74)	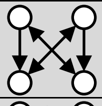
		M4-6552<br>(M3-36, 74)	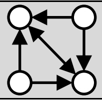

Table S11 The frequencies of consistent 4-node motifs on two microbial network with and without bioaugmentation. All calculations were manipulated by mfinder[7]: N(M4) is the number of motif in a network; N(**M4$_{random}$**) is the number of motif in a randomly generated network; Zscore is the Z test outcome form mfinder operations. Motif had a larger Zscore was noted as red.

	**Control**			**Bioaugmentation**		
	**N(M4)**	**N(M4$_{random}$)**	**Zscore**	**N(M4)**	**N(M4$_{random}$)**	**Zscore**
**Top 500**						
M4-404	-	-	-	-	-	-
M4-406	-	-	-	649	413.8±62.1	3.79
M4-908	-	-	-	-	-	-
M4-4682	-	-	-	-	-	-
M4-5004	-	-	-	-	-	-
**Top 1000**						
M4-404	1064	782.9±98.7	2.86	2350	1845.7±184.5	2.69
M4-406	1319	990±101	3.26	-	-	-
M4-908	72	30±12.4	3.38	250	93.8±23.1	6.76
M4-4682	205	98.4±20.5	5.20	229	126±27.3	3.78
M4-5004	70	11.4±5.7	10.35	78	29.3±12.2	3.98
**Top 1500**						
M4-404	1823	1274±136.7	4.02	2951	1980±220.8	4.40
M4-406	2493	2001.5±161.6	3.04	4141	3425.5±210.2	3.40
M4-908	133	71.3±19.1	3.24	311	90.3±24.3	9.08
M4-4682	316	170.9±32.9	4.41	504	255.2±41.9	5.93
M4-5004	157	39.1±13	9.04	203	46±16.1	9.73
**Top 2000**						
M4-404	2295	1740.6±169.7	3.27	3726	2776.8±247.9	3.83
M4-406	4048	2944.8±204.3	5.40	6702	5325.6±260.5	5.28
M4-908	284	143.4±30.1	4.67	633	150.2±32.4	14.89
M4-4682	348	186.8±42.5	3.79	715	385.9±60	5.49
M4-5004	352	79±19.9	13.71	272	83.2±23.5	8.05

Supplementary Figures:

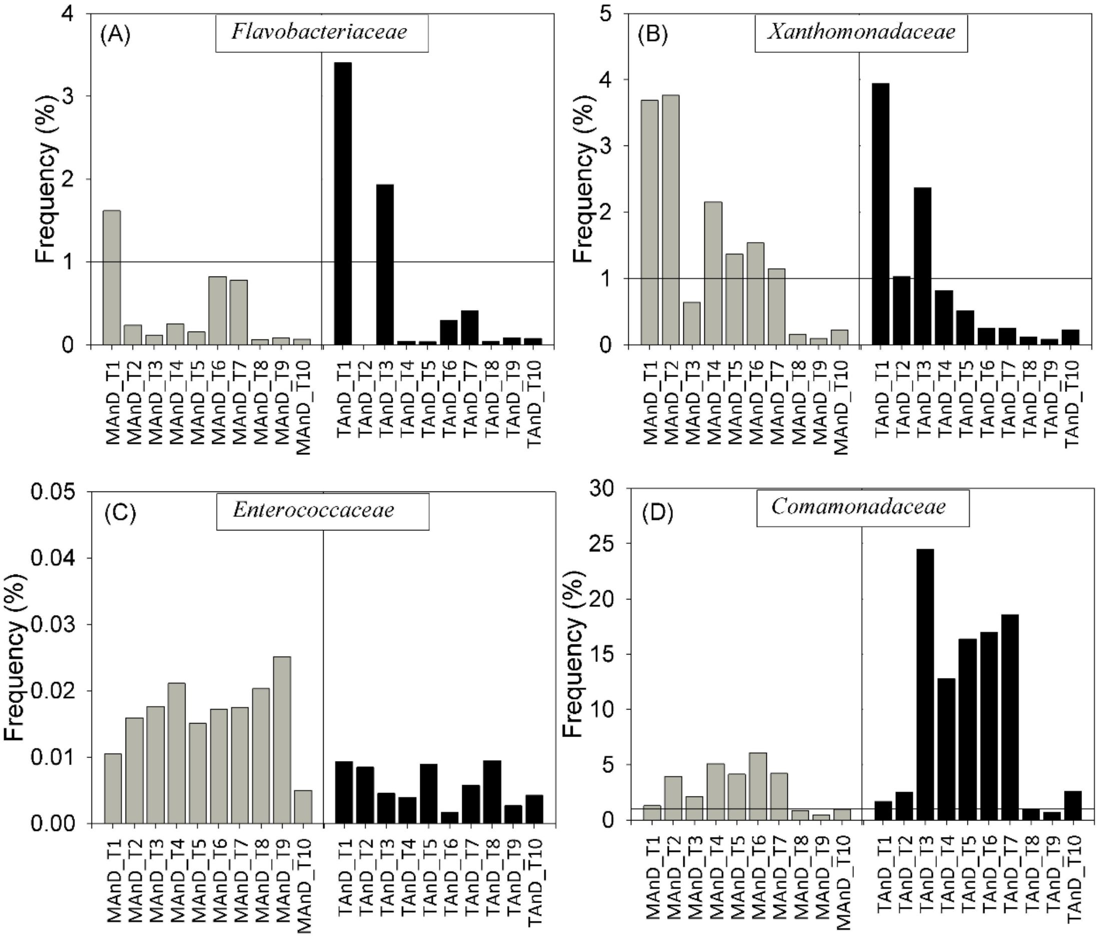

Figure S1 The low abundance of selected microbial families. They were (A) *Flavobacteriaceae*, (B) *Xanthomonadaceae*, (C) *Enterobacteriaceae*, and (D) *Comamonadaceae* from previous studies[1].

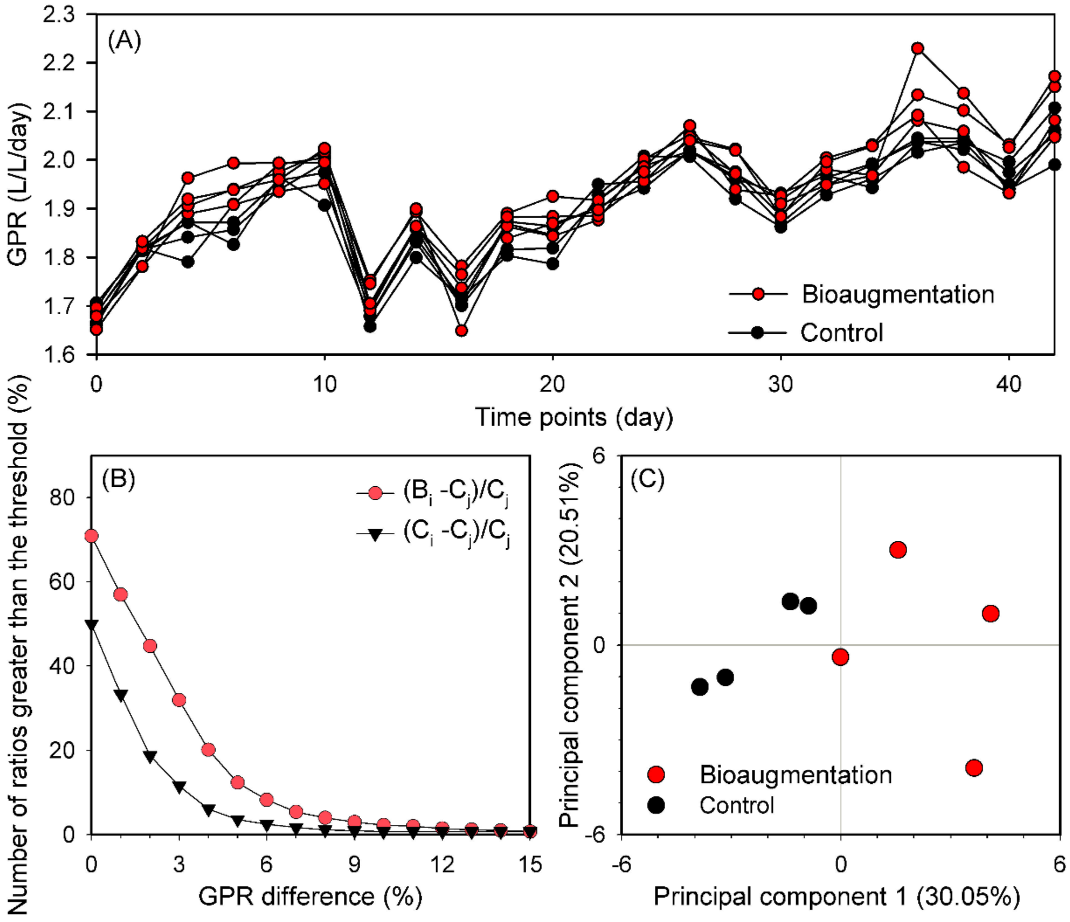

Figure S2 Reproducible biogas and methane production by eight independent CSTR reactors. B_i (or Cj) represented biogas yields (GPR) from four bioaugmented (or control) anaerobic digesters. (A) GPRs of eight anaerobic digesters before (black) and after (red) bioaugmentation were shown. (B) The distribution of GPR differences from bioaugmented anaerobic digesters was significantly different with that from the control (p=0.004; paired-samples Student's t test). (C) The PCA plot showed the separation of GPRs from control and bioaugmentation groups with 59.56% of variance explained by the first two principal components.

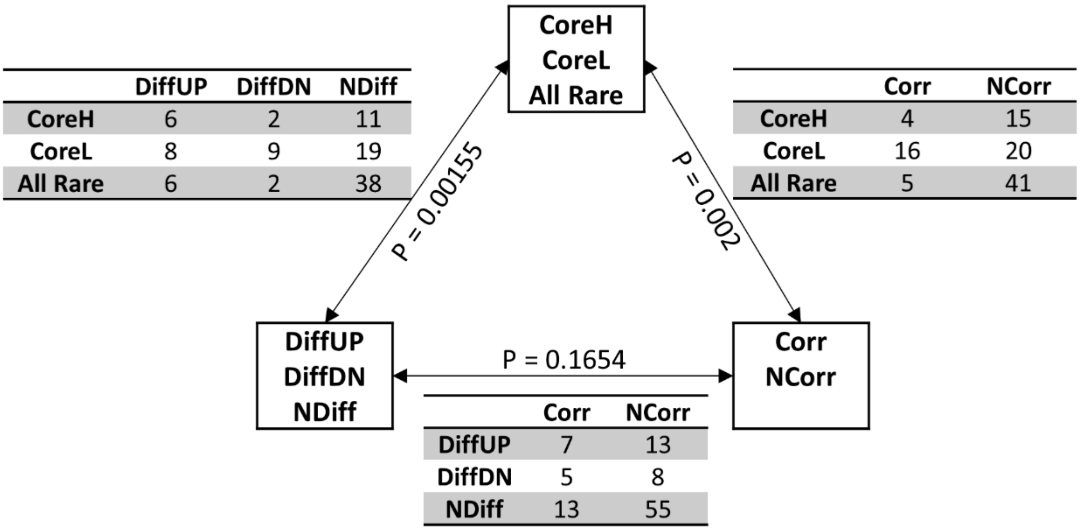

	DiffUP	DiffDN	NDiff
CoreH	6	2	11
CoreL	8	9	19
All Rare	6	2	38

	Corr	NCorr
CoreH	4	15
CoreL	16	20
All Rare	5	41

	Corr	NCorr
DiffUP	7	13
DiffDN	5	8
NDiff	13	55

Figure S3 The association in contingency tables between microbes partitioned by abundance level, differential abundance and abundance correlation under null hypothesis of absence of association based on chi-squared test.

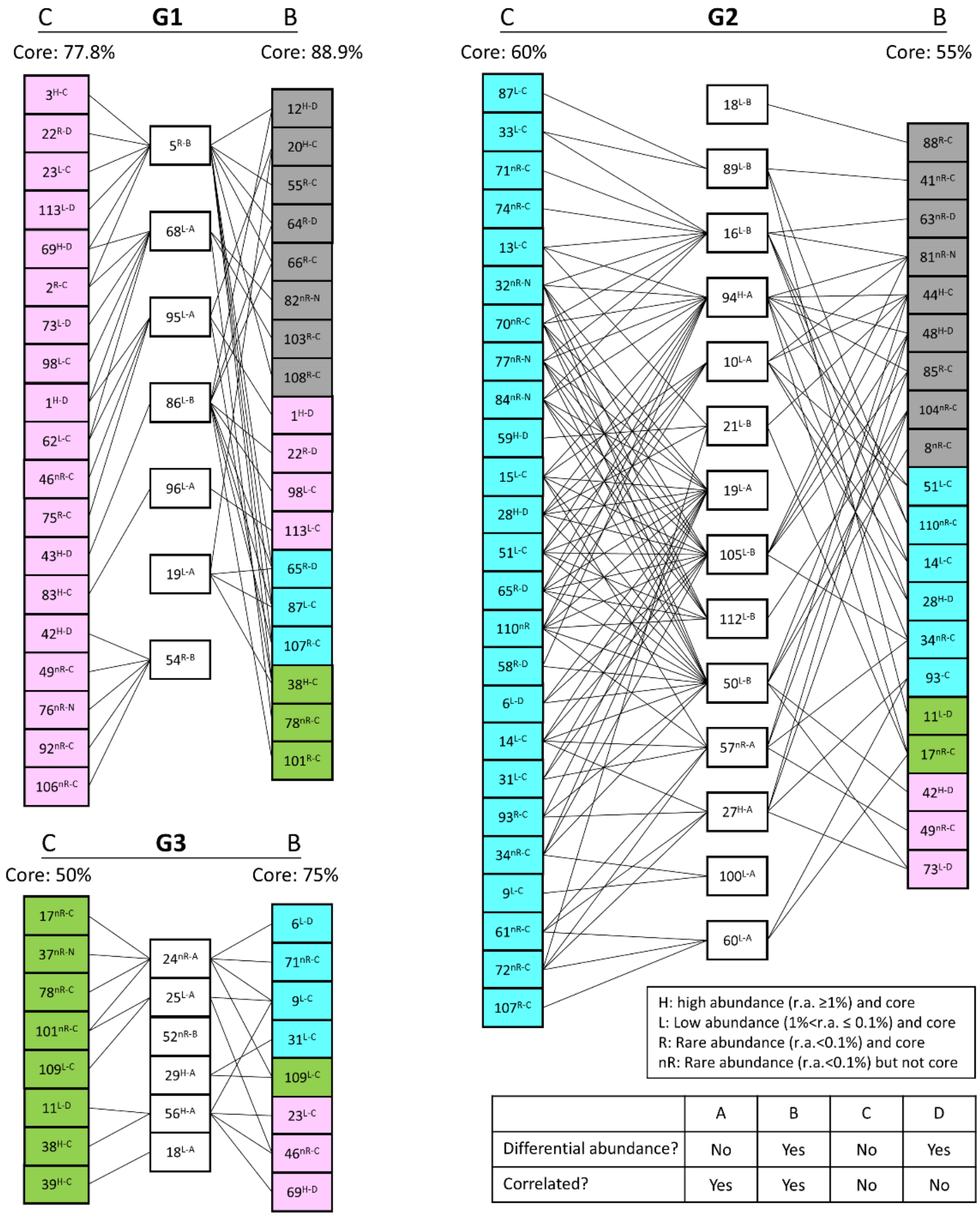

Figure S4 Three microbial clusters connected by microbes with correlated abundance patterns. The abundance characteristics of each microbe were denoted as H/L/R/nR for abundance level and A/B/C/D for combination of differential abundance status and correlated abundance patterns.

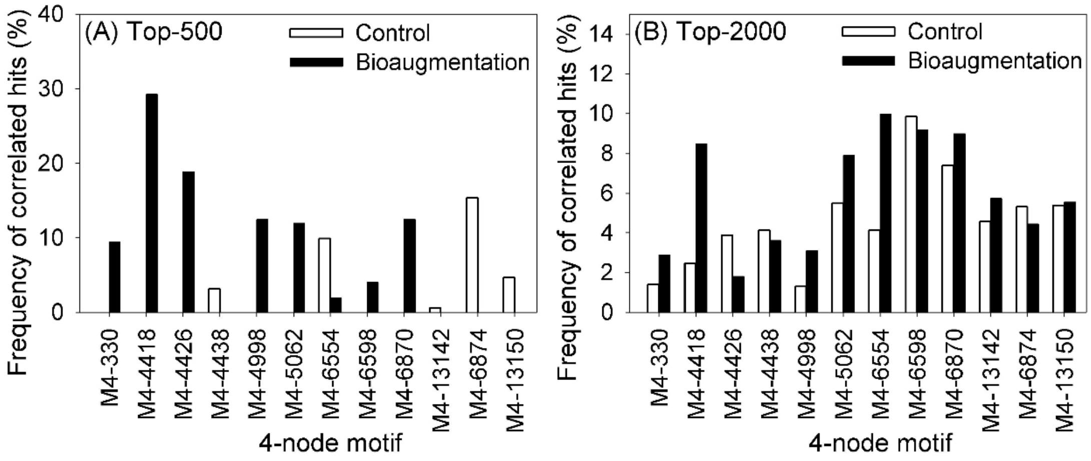

Figure S5 The frequency of correlated M3-98 pattern among all 4-node motifs with and without bioaugmentation. (A) The 500 and (B) 2000 strongest interactions were chosen.

Supplementary References:

1 Shaw, G. T., Liu, A. C., Weng, C. Y., Chou, C. Y. & Wang, D. Inferring microbial interactions in thermophilic and mesophilic anaerobic digestion of hog waste. *PloS one* **12**, e0181395, doi:10.1371/journal.pone.0181395 (2017).

2 Muto, A. *et al.* Modular architecture of metabolic pathways revealed by conserved sequences of reactions. *Journal of chemical information and modeling* **53**, 613-622, doi:10.1021/ci3005379 (2013).

3 Jochimsen, B. *et al.* Five phosphonate operon gene products as components of a multi-subunit complex of the carbon-phosphorus lyase pathway. *Proceedings of the National Academy of Sciences* **108**, 11393-11398 (2011).

4 Marvin-DiPasquale, M. *et al.* Methyl-mercury degradation pathways: a comparison among three mercury-impacted ecosystems. *Environmental Science & Technology* **34**, 4908-4916 (2000).

5 Compeau, G. & Bartha, R. Sulfate-reducing bacteria: principal methylators of mercury in anoxic estuarine sediment. *Appl. Environ. Microbiol.* **50**, 498-502 (1985).

6 Schloss, P. D. *et al.* Introducing mothur: open-source, platform-independent, community-supported software for describing and comparing microbial communities. *Applied and environmental microbiology* **75**, 7537-7541, doi:10.1128/AEM.01541-09 (2009).

7 Kashtan, N., Itzkovitz, S., Milo, R. & Alon, U. Efficient sampling algorithm for estimating subgraph concentrations and detecting network motifs. *Bioinformatics* **20**, 1746-1758, doi:10.1093/bioinformatics/bth163 (2004).
